# Differences in acoustic presence and vocal behavior of Spitsbergen’s bowhead whales under ice-covered and open-water conditions

**DOI:** 10.1038/s41598-025-25360-2

**Published:** 2025-11-18

**Authors:** Marlene Meister, Paul Keil, Karolin Thomisch

**Affiliations:** 1 Ocean Acoustics Group, Alfred Wegener Institute Helmholtz Centre for Polar and Marine Research, 27570 Bremerhaven, Germany; 2 Institute of Coastal Systems, Helmholtz Center Hereon, 21502 Geesthacht, Germany; 3 Data Analysis Department, German Climate Computing Center (DKRZ), 20146 Hamburg, Germany

**Keywords:** Baleen whales, Marine mammals, Acoustic ecology, Passive acoustic monitoring (PAM), Climate change, Migration, Ecology, Ecology, Ocean sciences, Zoology

## Abstract

Arctic-endemic bowhead whales (*Balaena mysticetus*) are facing extreme habitat changes, particularly due to ongoing sea-ice loss. This study compares acoustic presence and vocal behavior of bowhead whales at two ecologically distinct locations: (1) northwest of Svalbard at 81.5° N, a region that remains nearly ice-covered year-round, and (2) eastern Fram Strait at around 79° N, which is mostly ice-free throughout the year. To detect vocalizations in multi-year acoustic records, two Convolutional Neural Networks were trained. Acoustic presence periods were similar at both locations (October to April), but markedly higher and more continuous acoustic presence northwest of Svalbard indicates increased animal abundance. The frequent detection of song at this site further suggests that it serves as a winter breeding ground. Song diversity peaked in February, possibly reflecting animals arriving from north of Svalbard where sea ice retreated at that time. Regular acoustic detections between 2012 and 2023 in eastern Fram Strait show consistent seasonal presence of bowhead whales in this area. Our study contributes to assessing impacts of ongoing sea-ice decline on the Spitsbergen population and allows for more accurate predictions of future occurrence patterns. We provide new insights into intra-seasonal occurrence, thus support a holistic understanding of ecological characteristics of this population.

**Supplementary Information:**

The online version contains supplementary material available at 10.1038/s41598-025-25360-2.

## Introduction

As Arctic endemics, bowhead whales (*Balaena mysticetus*) are currently exposed to rapid and severe environmental shifts driven by climate change^1–5^. Among these shifts, sea-ice decline is of particular concern: bowhead whales regularly occur near the ice edge or deep within the ice, where they may benefit from increased prey availability and reduced predation by killer whales (*Orcinus orca*)^4,6–8^. The sharp decline in Arctic sea-ice cover over recent decades may lead to habitat loss, with bowhead whales having limited potential for northward range expansion due to reduced prey resources beyond the continental shelf edge^9^. Additional climate change-related threats include altered food-web dynamics, increased competition from temperate baleen whales extending their presence in Arctic feeding grounds, and intensified anthropogenic activities as the Arctic Ocean becomes more accessible for shipping^1–3,10^. Targeted conservation efforts are urgently needed but must be informed by detailed knowledge of distribution patterns, habitat use and migration behavior.

Despite having a rather restricted geographic range, bowhead whales, like most baleen whale species, undertake seasonal migrations^11–13^. The animals move between defined summering and wintering regions, with evidence of staggered migration based on sex, age and reproductive status^14–16^. Migration patterns differ between populations^14,17^ and current knowledge on migration is comparatively sparse for some populations, including the East Greenland–Svalbard–Barents Sea bowhead whale population (hereafter ‘Spitsbergen population’)^8,18^. Inhabiting waters between East Greenland and Franz Josef Land, this population was heavily reduced by past exploitation and remains critically low in numbers, with current estimates of only a few hundred individuals^19^. Data on the population’s migration behavior in the post-whaling era indicate that individuals travel to breeding areas at the northernmost latitudes of their range in autumn and disperse southward in spring—a pattern opposite to the typical north–south migration observed in most baleen whales^8,18^. A recent acoustic study improved understanding of population occurrence, identifying western Fram Strait and east of Svalbard as key overwintering and breeding grounds, while north of Svalbard may serve as a movement corridor^20^.

Under Arctic conditions, where visual surveys are often limited by sea ice and low light, acoustic studies play a key role to obtain insights on bowhead whale occurrence and migration patterns^21–23^. Bowhead whales are highly vocal and produce elaborate and diverse songs, presumably in a breeding context^13,24,25^. Songs follow a hierarchical structure, often compared to the more prominent songs of humpback whales, with individual units forming repeated phrases^24,25^. Bowhead whale singing behavior is likely linked to socio-sexual interactions (e.g., courtship behavior, rivalry)^13,25–27^, and might be specific to subgroups of individuals^27,28^. Therefore, song analysis offers a means to investigate bowhead whale behavior and occurrence, with song diversity possibly reflecting breeding intensity and changes in animal abundance.

**Fig. 1:**
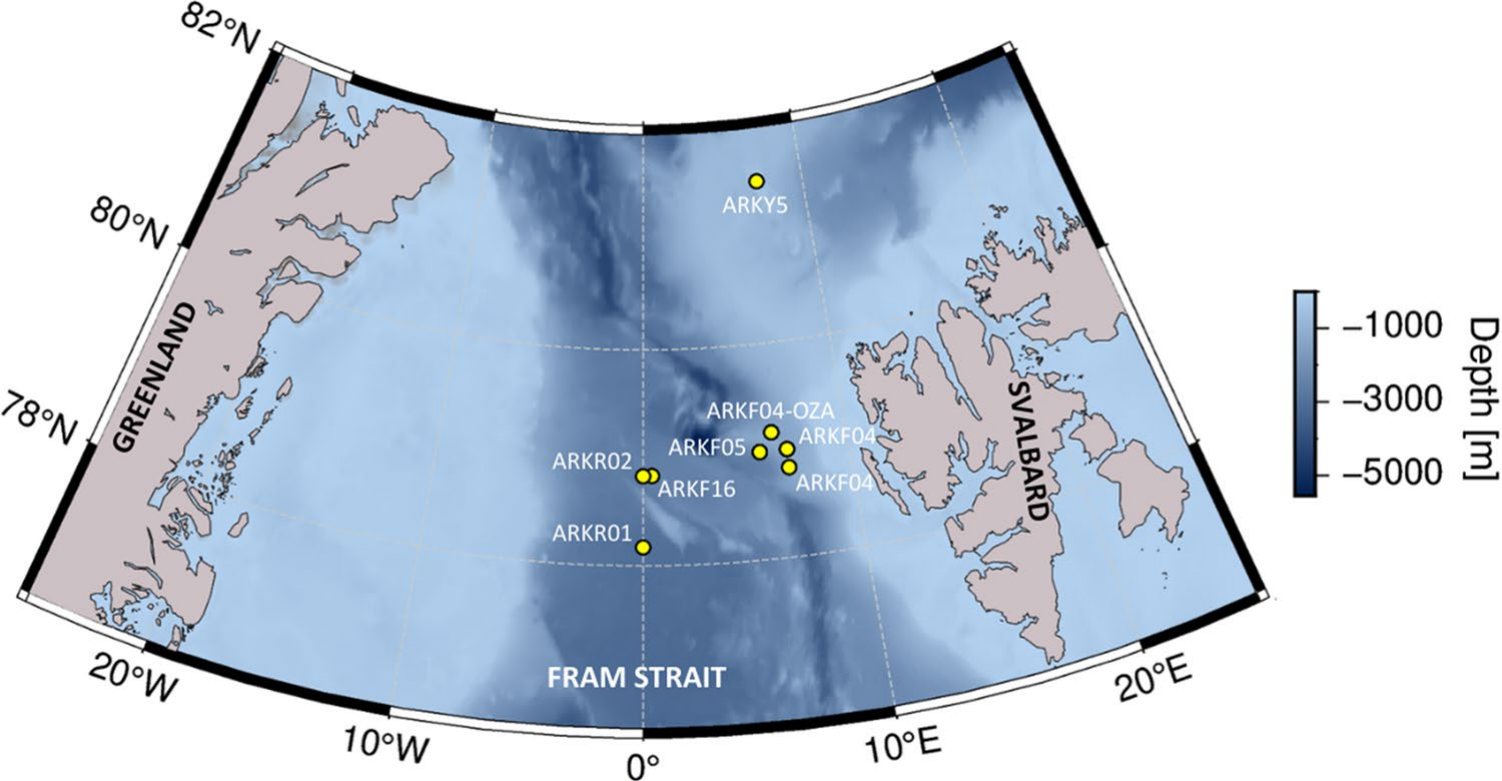
Recorder positions northwest of Svalbard and in Fram Strait. Bathymetry map was generated in PyGMT v0.9.0^31^ using the SRTM15 + V2.6 grid^32^.

Ongoing sea-ice decline will likely affect habitat suitability of bowhead whales, resulting in distributional and phenological shifts^4,9,29,30^. Our study contributes to assessing effects of sea-ice decline on bowhead whale occurrence based acoustic presence detected by two customized Convolutional Neural Networks (CNNs). We compared year-round acoustic presence at two ecologically contrasting locations: (1) northwest of Svalbard at 81.5° N, a proposed winter habitat that remains nearly ice-covered year-round^8,18^ and (2) eastern Fram Strait at 79° N, an open-water region sporadically visited by bowhead whales^22^ (Fig. 1). We further investigated vocal behavior and song occurrence to explore habitat use and potential intra-seasonal changes in animal occurrence. By that means, our study complements a previous acoustic effort focusing on western Fram Strait and the region north to east of Svalbard^20^, and supports a holistic understanding of population occurrence and migration behavior.

## Results

### CNN Performance

We trained two CNNs: one for data from northwest of Svalbard (CNN_No) and one for data from eastern and central Fram Strait (CNN_Ea_Ce). CNN_No achieved an overall validation performance of 81% sensitivity and a 0.7% false-positive rate, while CNN_Ea_Ce reached 83% sensitivity with a 0.7% false-positive rate for the validation dataset. Performance evaluation for individual recorders on an hourly basis showed reliable results, with sensitivity ≥ 80% and false-positive rates ≤ 1% after partial false-positive control (manual review of detections outside the typical bowhead whale calling period in winter and spring) for most recorders. Only one recorder (ARKF04-15_SV1026) showed decreased sensitivity (75%), likely due to a limited amount of available data with bowhead whale acoustic presence, which hindered a reliable performance evaluation. Performance values for individual recorders, along with a detailed description of CNN development, application, and performance evaluation, are provided in the Methods section.

### Acoustic presence

**Fig. 2:**
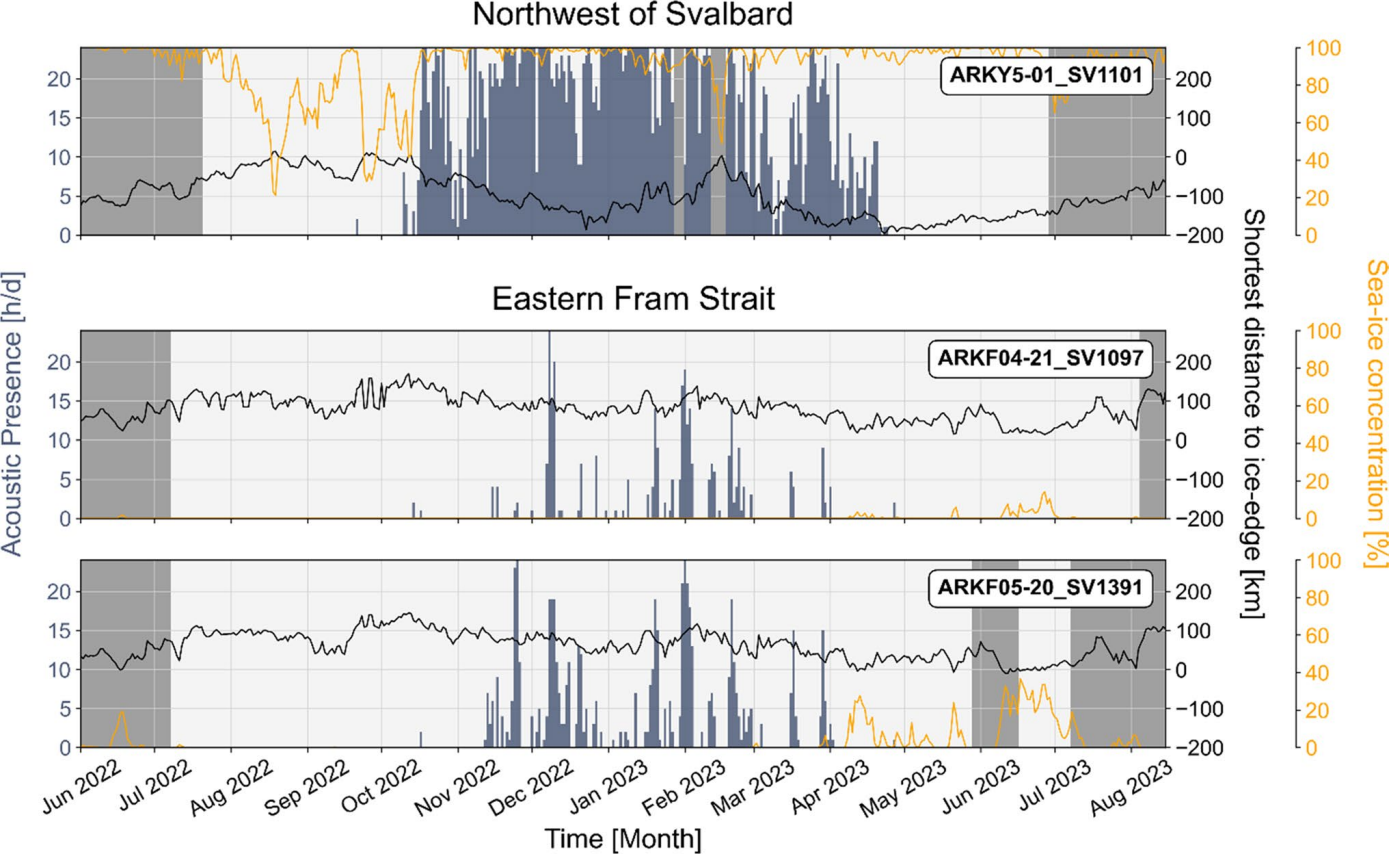
Acoustic presence of bowhead whales (hours per day) northwest of Svalbard and in eastern Fram Strait from 2022 to 2023. Black line shows shortest distance from recorder positions to the sea-ice edge, orange line shows sea-ice concentration averaged within a 35 km radius around each recorder position^22^. Negative values for distance to the sea-ice edge indicate that the recorder was located inside the ice, while positive values indicate it was located in open water. Gray shading denotes periods of missing data.

**Fig. 3:**
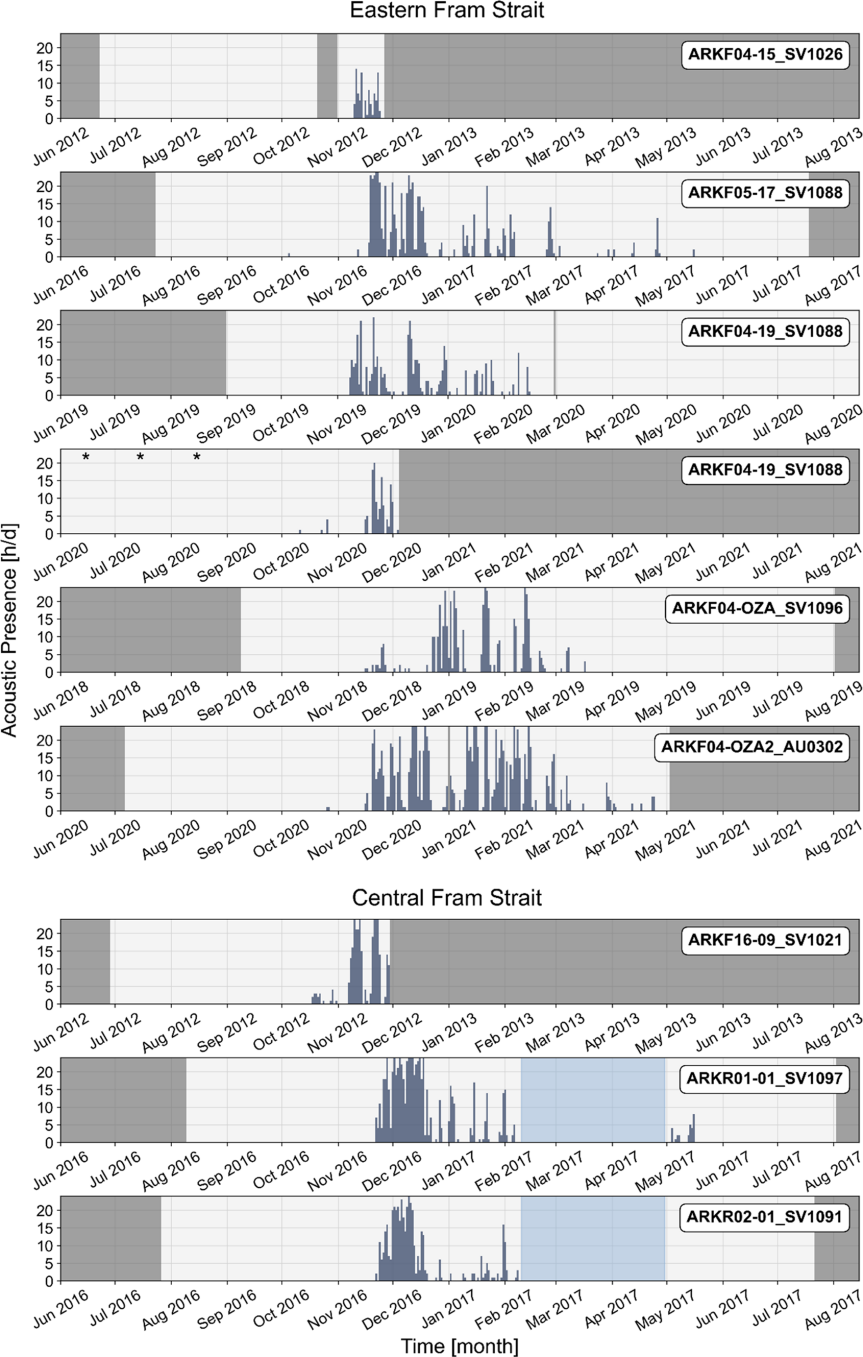
Acoustic presence of bowhead whales (hours per day) in eastern and central Fram Strait from 2012 to 2021. Gray shades indicate missing data. Due to the extended recording period of ARKF04-19_SV1088, two subplots were used for this recorder for display purposes. June, July, and August appear in both subplots and are marked with asterisks in the second to indicate repetition. Light blue shading indicates periods that were not analyzed due to prevailing sounds of breaking ice masking potential vocalizations.

Bowhead whales were detected at all locations and during all deployment years between autumn and spring (Figs. 2, 3). Acoustic data from the 2022/2023 deployment period revealed site-specific differences in bowhead whale acoustic presence patterns between northwest of Svalbard and eastern Fram Strait (Fig. 2). Northwest of Svalbard, sporadic vocalizations were first detected on September 21^st^, followed by continuous acoustic presence from October through April. Acoustic presence was generally high, reaching up to 24 h per day, though notable declines occurred in late October to early November, in March, and acoustic presence then ceased in late April. In total, this recorder documented 3,035 h of bowhead whale acoustic presence across 182 days. In eastern Fram Strait, regular acoustic presence occurred from November through March or early April (Fig. 2). It followed a bimodal pattern, with peaks in November to December and January to February, separated by periods of reduced acoustic presence. In total, ARKF04-21_SV1097 recorded 310 h of bowhead whale acoustic presence across 51 days, while ARKF05-20_SV1391 recorded 592 h across 82 days.

Sea-ice data indicate that the recorder northwest of Svalbard was ice-covered when bowhead whales were acoustically present, with sea-ice concentrations ranging from 60 to 100% and shortest distance to the ice edge ranging from 10 to 196 km (Fig. 2). In contrast, both recorders in eastern Fram Strait were located in open water when bowhead whales were acoustically present, with shortest distance to the ice edge ranging from 18 to 150 km (Fig. 2).

Acoustic data from earlier deployment periods (2012 to 2021) in eastern and central Fram Strait showed interannual variability, with the main period of bowhead whale acoustic presence typically occurring from November to February or March (Fig. 3). In two recorders (ARKF04-19_SV1088 and ARKF16-09_SV1021), vocalizations were first detected as early as October. Additionally, three recorders (ARKF05-17_SV1088, ARKF04-OZA2_AU0302, and ARKR01-01_SV1097) documented sporadic vocalizations extending into April or May. Comparisons between recorders did not indicate a consistent pattern but instead revealed multiple irregular peaks in acoustic presence, reaching up to 24 h per day, interspersed with periods of low or no acoustic presence.

### Vocal behavior and song analysis

**Fig. 4:**
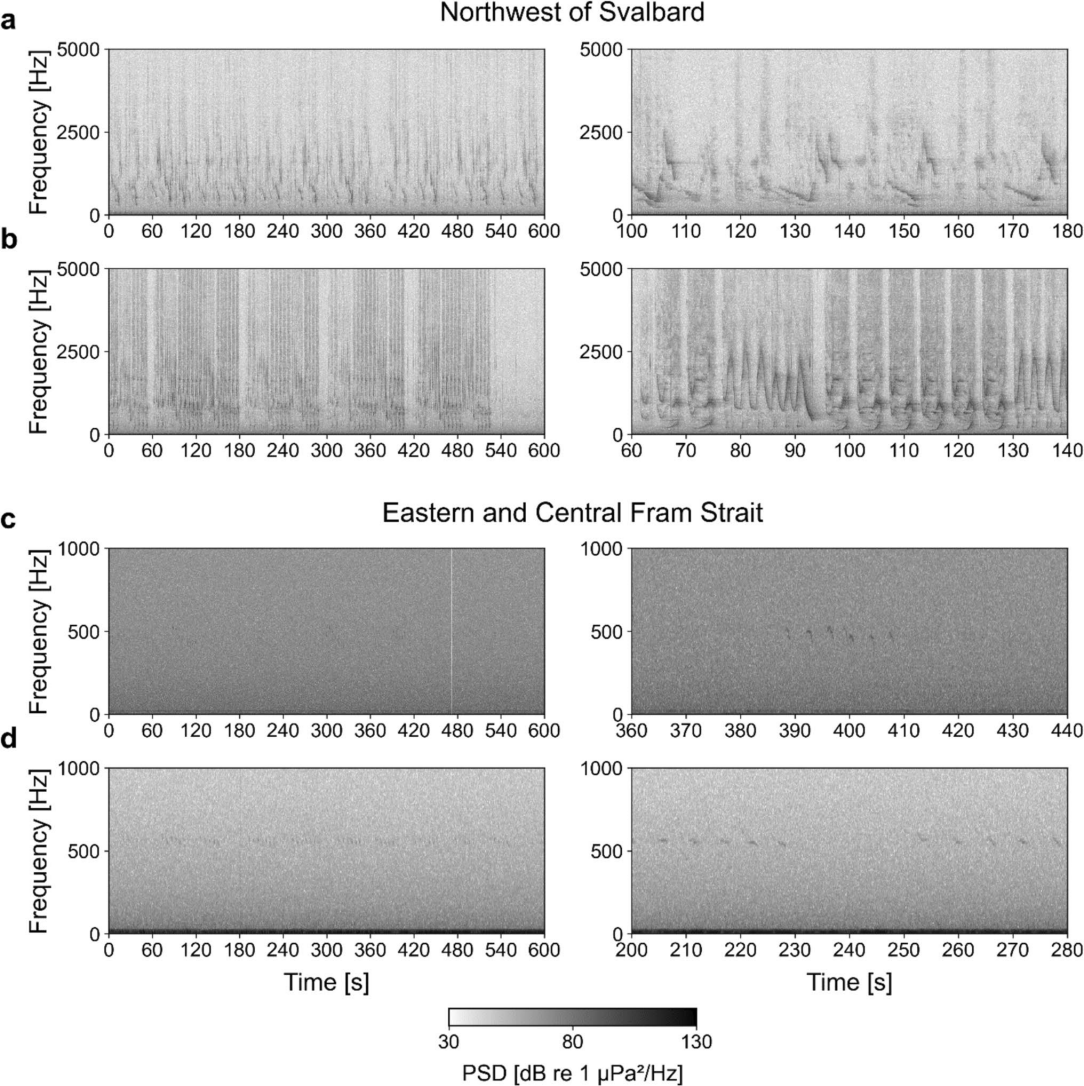
Bowhead whale vocalizations recorded northwest of Svalbard (a, b: ARKY5-01_SV1101 on 21.11.2022 and 04.01.2023) and in Fram Strait (c: ARK16-09_SV1021 on 20.11.2012; d: ARKF04-21_SV1097 on 20.02.2023), illustrating differences between sites. Northwest of Svalbard, detected vocalizations were primarily part of complex songs, whereas in Fram Strait vocalizations were either single calls or (part of) calling sequences, often at low amplitude levels. Panel a shows Song 1 and panel b shows Song 4 (see Fig. 5). PSD = Power Spectral Density.

During manual review of the sample dataset, we noted the presence of simple calls, calling sequences, and song, according to the definition of Stafford et al.^33^. Vocalizations differed markedly between northwest of Svalbard and all Fram Strait positions (Fig. 4): Northwest of Svalbard, vocalizations were primarily part of song, arranged in repeated sequences with complex frequency modulations and regularly containing energy above 2 kHz. Harmonics and the simultaneous occurrence of two distinct sounds (two-voiced singing) were also common. In contrast, vocalizations in Fram Strait were simple frequency-modulated (FM) calls mainly below 1 kHz. These occurred alone or in bouts, suggesting they were single calls or part of calling sequences. Amplitude levels were comparatively low, and often only parts of the calls or sequences were visible. No song as defined by Stafford et al.^33^ was detected at our Fram Strait positions.

**Fig. 5:**
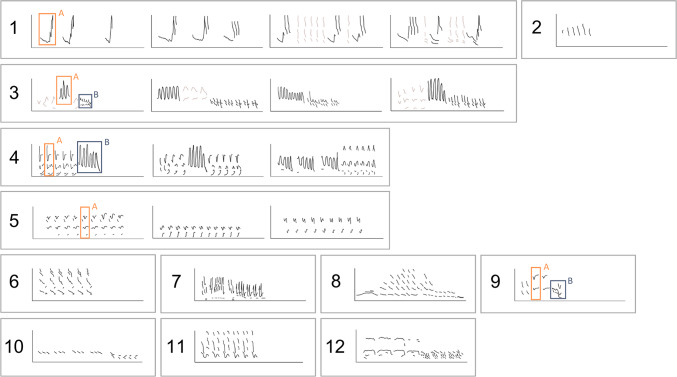
Sketches representative of different songs recorded northwest of Svalbard from October 2022 to April 2023. X-axes indicate time from 0 to 600 s, y-axes indicate frequency from 0 to 2500 Hz. Distinct variations in unit structure and arrangement were observed for songs 1, 3, 4, and 5. Song 1 and 3 contained irregularly occurring units (brown).

During song analysis in the northwest Svalbard data, we identified 12 different songs (Fig. 5, see Supplementary Fig. S2–S30 for exemplary spectrograms of each song). For Song 2, the sequence structure was faint and never fully visible, inhibiting a clear distinction as to whether it was a song or a calling sequence. Within four songs (Songs 1, 3, 4, and 5), we observed notable variation in the structures of the same units.

We observed gradual transitions from one song to another, particularly evident for Song 1 (Fig. 5). Over time, the A-unit of Song 1 gradually split into two parts, eventually forming the A- and B-units of Song 3. Song 4 likely evolved from Song 3, maintaining its unit A; Song 5 likely evolved from Song 4, maintaining its unit A; and Song 9 likely evolved from Song 3, maintaining its unit B.

**Fig. 6:**
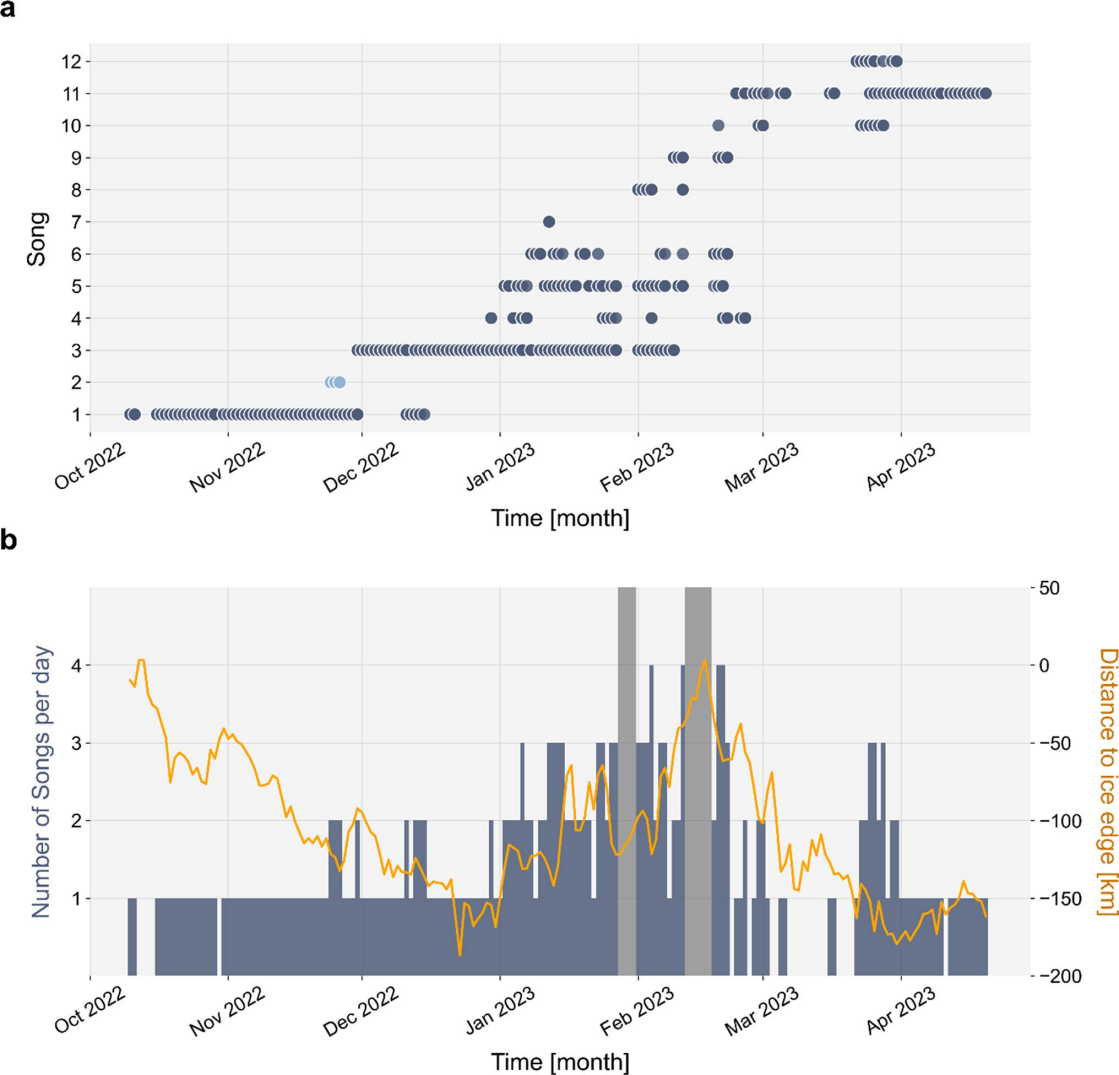
(**a**) Daily song occurrence northwest of Svalbard from October 2022 to April 2023. For Song 2 (indicated in lighter blue), full sequence structure was never visible in the spectrograms viewed. (**b**) Number of unique songs per day and shortest distance to the sea-ice edge northwest of Svalbard.

Songs appeared and subsequently disappeared in succession with partial temporal overlap (Fig. 6). After their first occurrence, most songs were not detected continuously but appeared intermittently, with their presence interrupted by periods of several days or even weeks without detection. The most frequently detected songs were Song 3 (65 days) and Song 1 (53 days).

The number of unique songs per month increased gradually from one in October to eight in February, then decreased to three in March and one in April (Fig. 6a). Similarly, the number of unique songs per day peaked in February (up to four songs per day), coinciding with a sea-ice retreat that brought the ice-edge close to the recorder (Fig. 6b).

## Discussion

Our findings suggest increased bowhead whale occurrence in the ice-covered region northwest of Svalbard compared to the open-waters of eastern Fram Strait, as indicated by higher and more continuous acoustic presence (Fig. 2). While site-specific differences in acoustic presence patterns might also be influenced by changes in calling behavior^33^, the characterization of the area northwest of Svalbard as a preferred habitat is consistent with tracking data^8,18^. We further document distinct differences in vocal behavior between sides, with song detected exclusively and persistently northwest of Svalbard, suggesting this site to serve as a breeding ground (Fig. 5, 6). The song analysis showed that song diversity peaked in February, probably reflecting increased animal abundance. Our findings are based on detections from two custom-built CNNs that demonstrated strong validation performance (> 80% sensitivity, < 1% false positive rate, see Methods section for detailed information on CNN performance). Partial false-positive control of detections outside the usual bowhead whale calling period in winter and spring further supports the reliability of the results.

Northwest of Svalbard, we detected song throughout the calling season (from October to April) indicating reproductive behavior among bowhead whales at this site. This aligns with the migration pattern of the Spitsbergen population, which moves towards northern breeding grounds in autumn and disperses from there in spring^8,18^. It further adds to previous identifications of breeding grounds in western Fram Strait at approximately 79° N (ca. 380 km from our location)^20,21,33–35^ and east of Svalbard (ca. 495 km from our location)^20^. The recorder northwest of Svalbard was mostly covered by pack ice, at times located nearly 200 km from the ice edge (Fig. 2). Simultaneous acoustic detections of bowhead whale confirm that recorded animals were often located (deep) within the pack ice. Spitsbergen’s bowhead whales have been reported to show an affinity for dense sea-ice during winter^8,20,21^, which might be linked to the presence of polynyas and flaw-lead systems^18^. Furthermore, sea-ice might function as a refuge from predatory killer whales, with ongoing sea-ice decline likely promoting killer whale presence in Fram Strait and reducing available refuge for bowhead whales^7,36,37^. This might result in greater predation pressure and reduced reproductive success^7,37^, highlighting the ecological importance of sea-ice for the Spitsbergen population.

Overall, we identified 12 songs northwest of Svalbard (Fig. 5), notably fewer than the 39 to 76 songs per season reported in a previous study from western Fram Strait^35^. The difference between studies may reflect variation in animal occurrence, behavior, or interannual changes, but methodological differences likely also played a role. Manual song classification is highly subjective, and manual reviewers may apply different thresholds for distinguishing between songs^38^. In our study, we observed gradual song development, which made it challenging to decide whether a song was new or a variation of a previous one. To stay consistent, we distinguished only between songs with markedly distinct unit structures and arrangements, which likely reduced the total number of identified songs in comparison to other studies.

The observed gradual transitions between certain songs may indicate that individuals modified their singing behavior over time and, as a result, produced more than one song type during the season. The detection of these transitional songs over several months would then suggest that singing individuals remained near the recording site for an extended period. However, it is unclear whether individual bowhead whales sing different songs in one season^24,33,35^. Since bowhead whale share song among individuals^13,26–28,38^, these gradual changes could also reflect song transmission between animals, as also observed in humpback whales^27,39^.

We propose that higher song diversity in February 2023 may reflect an increase in animal abundance, although the relationship between song diversity and animal abundance is not well understood^26–28,35^. Supporting this hypothesis, the peak in song diversity coincided with a retreat of sea ice north of Svalbard, creating a large open-water area that extended partly beyond 82° N and brought the ice edge closer to the recorder (Fig. 6, Supplementary Fig. S1). Sea-ice conditions in this region exhibit pronounced variability, with periods of reduced ice concentration linked to the presence of warm Atlantic water, elevated air temperatures, and prevailing southerly winds^40,41^. It is possible that open-water conditions north of Svalbard reduced bowhead whale habitat suitability in February, causing whales to follow the receding ice toward northwest of Svalbard and increase the number of individuals at our recording location. The resulting local increase in individuals could have contributed to the observed peak in song diversity, with arriving singers introducing new songs^28^ or adding variation to existing ones. Additionally, the peak in song diversity could also reflect more varied singing behavior of individual whales. Song is thought to be produced by males to attract females or to dominate rivals^13,24^, and song novelty may provide reproductive advantage to the singer^24,35^. Thus, higher song diversity may indicate increased breeding activity at the recording site. This, in turn could also be linked to increased animal abundance: a greater number of singers may stimulate competitive displays, as shown in humpback whales, where males switched more frequently between phrases when a second male started singing^42^.

Our findings suggest that bowhead whales occur less frequently in the open waters of eastern Fram Strait than in the ice-covered region northwest of Svalbard, but seasonal detections of their vocalizations in every year from 2012 to 2023 show that eastern Fram Strait is still regularly used. Acoustic presence of bowhead whales was previously recorded at this area for two seasons^22^, however, tracking studies, with up to 13 tracked individuals, so far have not provided evidence of individuals entering the area^8,18^. Vocalizations recorded in eastern and central Fram Strait were relatively faint, likely due to the calling animals being located at a distance from the recorders. However, we are confident that the recorded sounds were still produced locally and not transmitted from western or northern Fram Strait regions, due to a previous study showing discrete acoustic sampling of bowhead whales for two recorders in Fram Strait being 95 km apart^33^. Furthermore, the distinct vocal behavior observed, characterized by the predominant occurrence of calls and calling sequences throughout the breeding season, has previously been described for eastern and central Fram Strait^22,33^. The absence of song suggests that this open-water area does not serve as a breeding ground, but is more likely intermittently visited by animals, with vocalizations probably used to aid navigation or to contain cohesion and contact between individuals^13,33,43^. Furthermore, the characteristic calling behavior may reflect the presence of only certain sex or age cohorts^22^. The reason for bowhead whales visiting eastern Fram Strait may be related to feeding or transit; however, the ecological significance of this area for the Spitsbergen population remains uncertain.

By integrating our findings with previous studies, we contribute to a more holistic understanding of occurrence and migration of Spitsbergen’s bowhead whales. Animals move towards northern breeding grounds in autumn^8,18^ and breed during winter in sea-ice covered regions including northwest of Svalbard (our study), western Fram Strait^20,21,33–35^ and east of Svalbard^20^. Regions with little or unstable sea-ice cover, including eastern Fram Strait (this study) and north of Svalbard^20^, appear to serve a different ecological function, likely acting as migratory corridors^20,22^, as habitats for specific sex or age cohorts^22^, or provide feeding opportunities. Our finding of increased song diversity northwest of Svalbard in February 2022 may reflect higher animal abundance at the recording location, probably due to animals arriving from north of Svalbard where sea ice retreated at that time. This could imply that Spitsbergen’s bowhead whales track shifting ice edges to breed in the pack ice, suggesting that ongoing sea-ice decline will lead to distributional shifts during winter towards remaining sea-ice areas. Future collaborative long-term efforts, such as acoustic transect or tracking studies, will aid verification of this hypothesis. Nevertheless, potential northward habitat expansion might be eventually constrained by reduced prey availability beyond the continental shelf edge^9^. Hence, remaining sea-ice habitats in the Fram Strait region might be of particular importance as winter breeding habitats for the Spitsbergen population.

## Conclusion

In our study, we used custom-built CNNs that successfully detected bowhead whale calls, calling sequences and song. We used a file-based approach, which made training data labelling efficient, thus allowing easy adaptation of the CNNs to other regions. To ensure reliable detection in other regions, we recommend training CNNs with representative data that includes bowhead whale vocalizations and potential false-positive sources specific to each time and location, or applying manual post-processing. 

We combined acoustic presence data with a song analysis, which proved highly useful for gaining more nuanced insights into habitat use and migration behavior. Our findings help to assess impacts of sea-ice decline on the Spitsbergen population and to fill knowledge gaps on its intra-seasonal occurrence.

For a more comprehensive understanding of the occurrence and migratory behavior of Spitsbergen’s bowhead whales, international collaboration is needed to standardize and combine PAM efforts across different locations. Additionally, integrating diverse data types (e.g., acoustic, tagging, observational, and genetic) is essential to gain a holistic view of the ecological and demographic characteristics of the population. Internationally coordinated frameworks, such as the proposed Arctic-Atlantic Distributed Biological Observatory (DBO, https://arcticpassion.eu/adbo/), will foster cross-disciplinary collaboration in the Atlantic sector of the Arctic Ocean (and beyond) and thereby contribute to a pan-Arctic observational network.

## Methods

### Data collection

We investigated bowhead whale acoustic occurrence and behavior using acoustic data collected between 2012 and 2023 at eight different recording positions (seven positions in eastern/central Fram Strait at around 78/79° N, one northwest of Svalbard at 81.5° N; Fig. 1). Data were collected through the Ocean Observing System FRAM (Frontiers in Arctic Marine Monitoring)^44^ by the Alfred Wegener Institute Helmholtz Centre for Polar and Marine Research (AWI). Passive acoustic recorder of the type Sono.Vault (Develogic GmbH, Hamburg, Germany) and AURAL recorder (Autonomous Underwater Recorder for Acoustic Listening; Model 2, Multi-Électronique, Rimouski, QC, Canada) were deployed and recovered from research vessels, including RV Polarstern^45^. Sampling rate was between 5.333 kHz and 96 kHz and data were stored as 10-min .wav files (Table 1). Raw audio data were processed following AWI’s Standard Operating Procedures (SOPs) for passive acoustic monitoring data^46,47^. Data quality control was performed by inspecting spectrograms via the Open Portal to Underwater Soundscapes (OPUS, https://opus.aq), according to AWI’s SOPs^48^. Data are available via the data repository PANGAEA (https://www.pangaea.de)^49–55,56^.

**Table 1:** Locations and recording settings of passive acoustic recorders deployed in Fram Strait between 2012 and 2023.

Mooring ID	Recorder Serial Number	Latitude [°]	Longitude [°]	Deployment period	Recording period	Water depth [m]	Recorder depth [m]	Sample rate [kHz]	Effective bandwidth [Hz]	Duty cycle
ARKY5-01	SV1101	81.50	7.15	07.2022–06.2023	07.2022–06.2023	477	301	48	8–24,000	continuous
ARKF04-15	SV1026	78.83	7.00	06.2012–06.2015	06.2012–11.2012	1420	743	5.333	8–2666	continuous
ARKF04-OZA	SV1096	79.17	6.33	09.2018–07.2020	09.2018–08.2019	1418	836	48	8–24,000	continuous
ARKF04-19	SV1088	79.00	7.00	08.2019–06.2021	09.2019–12.2020	1218	805	48	8–24,000	10 min per 20 min
ARKF04-OZA2	AU0302	79.17	6.33	07.2020–07.2022	07.2020–05.2021	1422	300	32	10–16,000	10 min per h, continuous
ARKF04-21	SV1097	79.00	7.00	07.2022–08.2023	07.2022–08.2023	1224	300	48	8–24,000	continuous
ARKF05-17	SV1088	79.00	5.67	07.2016–09.2018	07.2016–07. 2017	2100	808	48	8–24,000	continuous
ARKF05-20	SV1391	79.00	5.67	07.2022–07.2023	07.2022–07.2023	2087	309	96	8–48,000	continuous
ARKF16-09	SV1021	78.83	0.43	06.2012–09.2014	06.2012–11.2012	2525	800	5.333	8–2666	continuous
ARKR01-01	SV1097	78.17	0.00	08.2016–07.2018	08.2016–08.2017	3013	752	48	8–24,000	continuous
ARKR02-01	SV1091	78.83	0.00	07.2016–07.2018	07.2016–07.2017	2587	806	48	8–24,000	continuous

### Data analysis

The dataset consisted of 3,541 days of passive acoustic recordings, analyzed at the file level (10-min intervals) to detect bowhead whale presence using custom-built CNNs^57–59^. In a first step, we created a sample dataset by selecting the 5^th^, 10^th^, 15^th^, 20^th^, and 25^th^ day of each month, whenever available, from data recorded by nine recorders (Supplementary Table S1). To reduce computational load meanwhile model training, we excluded ARKR01-01 and ARKR02-01 from the training dataset. We chose these recorders as (i) the training dataset already included data from ARKF05-17, which covered a similar time period as ARKR01-01 and ARKR02-01, and (ii) both recorders had been previously analyzed for bowhead whale vocalizations by Thomisch et al.^22^ (using the Low-Frequency Detection and Classification System (LFDCS)^60^, allowing us to verify the CNN’s predictions for these recorders. We then manually analyzed this sample dataset in Raven Pro 1.6.4 (Bioacoustics Research Program, Cornell Lab of Ornithology)^61^ using the following spectrogram settings: 5.333 kHz sample rate (window size: 2,800 samples, Hann window, DFT size: 4,096 samples, hop size: 1,400); 32 & 48 kHz sample rate (window size: 18,000 samples, Hann window, DFT size: 32,768 samples, hop size: 9,000); and 96 kHz sample rate (window size: 40,000 samples, Hann window, DFT size: 65,536 samples, hop size: 20,000). Files were analyzed in the 8 to 1000 Hz range, with at least one detected vocalization indicating acoustic presence per 10 min, and days with detections were re-examined manually in Raven Pro in the 8 to 5000 Hz range to assess high-frequency components.

### Convolutional neural network

To analyze acoustic data from northwest of Svalbard, we trained a custom-built sequential CNN (CNN_No) using data from both northwest of Svalbard and eastern/central Fram Strait (Supplementary Table S1). For eastern/central Fram Strait, we trained a separate CNN (CNN_Ea_Ce) using only data from eastern/central Fram Strait (Supplementary Table S1). This separation into two models was based on observations from manual analysis of the sample dataset, which showed that bowhead whale vocalizations and environmental sounds (e.g., cryogenic sounds) differed markedly between northwest of Svalbard and eastern/central Fram Strait. Due to limited training data from northwest of Svalbard, CNN_No also included recordings from the eastern/central Fram Strait. In contrast, model performance for individual recorders in CNN_Ea_Ce improved when excluding data from northwest of Svalbard. The model code is freely available at: https://gitlab.awi.de/oza-sound-detectors/cnn_file_detection/.

#### Input preparation

We downsampled and normalized all audio data to a consistent sample rate of 5 kHz across all recorders. Using the Python package librosa, we generated spectrograms for the 50 to 1000 Hz frequency range (window size: 2,800 samples, Hann window, DFT size: 4,096 samples, hop size: 1,400). Each spectrogram covered a length of 600 s and missing values were filled with zero-padding. Spectrograms with labels derived from manual analysis of the sample dataset, as well as unlabeled inference data, were saved in Zarr format for each recorder, enabling efficient loading of data from disk during training.

#### Model architecture

CNNs are commonly used for image classification, including spectrogram classification^62^. We designed an architecture for binary image classification tasks, consisting of 7 convolutional layers with kernel sizes of [7, 5, 5, 3, 3, 3, 3], a stride of 1, and max-pooling layers of size 2. Dropout at a rate of 0.2 was applied after each layer^63^, and the channel sizes progressively increased from 1 to 100 ([1, 5, 10, 20, 40, 60, 80, 100]). After passing through the convolutional layers, the feature map had a shape of (11 × 32 × 100). Average pooling then reduced it to (1 × 1 × 100) before it was fed into the classifier, which had two fully connected layers with 128 and 32 neurons, followed by the output layer. In total, this architecture amounted to approximately 350,000 trainable parameters.

#### Model training

For model training, Zarr files containing labelled data from the respective recorders were divided into two subsets: 80% for training (CNN_Ea_Ce: 39,684 files; CNN_No: 46,012 files) and 20% for validation (CNN_Ea_Ca 9,921 files; CNN_No: 11,503 files). The input data were standardized using Z-score normalization. Class weights were used during training to account for differences in class frequency, as part of the overall training configuration (Supplementary Table S2). Validation metrics, including sensitivity and false positive rate, were recorded throughout training. A model was saved if sensitivity reached at least 0.8 and the false positive rate was below 0.01, with further improvements in false positive rate updating the best model. Early stopping with a patience of 15 epochs was applied, terminating training if no new best model was saved within this period. As a result, CNN_Ea_Ce was saved at epoch 76 and CNN_No at epoch 40. Training took approximately 12 (CNN_Ea_Ce) and 9 (Cnn_No) hours on an A100 NVIDIA GPU. 

#### Model predictions

Model output probabilities were converted to binary labels, with values above 0.5 classified as positive and those below as negative. We then performed a partial false-positive control by manually reviewing all detections from March 1^st^ until the first true detection in November, as they fell outside the typical bowhead whale calling period in winter and spring^21,22^.

#### Model performance

CNN_No achieved a validation performance of 81% sensitivity with a 0.7% false-positive rate, while CNN_Ea_Ce achieved 83% sensitivity with 0.7% false-positive rate. Hourly performance for individual recorders is provided in Table 2.

**Table 2:** Performance metrics for individual recorders included in the sample dataset.

Recorder	CNN	Validation score [Sensitivity/FP-rate]	Hourly performance (Before FP-control) [Sensitivity/FP-rate]	Hourly performance (After FP-control*) [Sensitivity/FP-rate]	Total amount of hours	#Hours before FP-control [TP/TN/FP/FN]	#Hours after FP-control* [TP/TN/FP/FN]
ARKY5-01_SV1101	CNN_No	0.87/0.0417	0.95/0.10	0.94/0.01	1320	537/677/77/29	537/745/9/29
ARKF04-15_SV1026	CNN_Ea_Ce	0.50/0.0014	0.71/0.00	0.71/0.00	588	5/580/1/2	5/581/0/2
ARKF04-OZA_SV1096	CNN_Ea_Ce	0.61/0.0116	0.80/0.02	0.80/0.01	1296	48/1212/24/12	48/1221/15/12
ARKF04-19_SV1088	CNN_Ea_Ce	0.82/0.0056	0.85/0.01	0.85/0.00	1800	97/1663/23/17	97/1678/8/17
ARKF04-OZA2_AU0302	CNN_Ea_Ce	0.85/0.0016	0.89/0.01	0.89/0.00	1176	159/990/8/19	159/995/3/19
ARKF04-21_SV1097	CNN_Ea_Ce	0.89/0.0045	0.90/0.02	0.90/0.01	1536	47/1461/23/5	47/1471/13/5
ARKF05-17_SV1088	CNN_Ea_Ce	0.76/0.0117	0.92/0.02	0.91/0.01	1416	92/1296/20/8	92/1302/14/8
ARKF05-20_SV1391	CNN_Ea_Ce	0.91/0.0046	0.91/0.02	0.91/0.01	1359	106/1219/24/10	106/1236/7/10
ARKF16-09_SV1021	CNN_Ea_Ce	0.82/0.0045	0.98/0.01	0.98/0.01	596	43/548/4/1	43/549/3/1

Hourly performance was calculated by assigning each file to the full hour in which it started.

TP = true positives, TN = true negatives, FP = false positives, FN= false negatives.

*A partial FP-Control was conducted each year from March 1^st^ until the first confirmed detection in September.

A comparison of our CNN detections with previous analyses of five recorders using the Low-Frequency Detection and Classification System (ARKF04-15_SV1026, ARKF16-09_SV1021, ARKF05-17_SV1088, ARKR01-01_SV1097, ARKR02-01_SV1091)^22,59^ revealed highly similar detected acoustic presence patterns for both methods. This comparison included two unseen recorders, i.e. they were not included in the CNN training (ARKR01-01_SV1097 and ARKR02-01_SV1091). To further evaluate performance on unseen data, we trained an additional CNN using data from six recorders (ARKF04-15_SV1026, ARKF16-09_SV1021, ARKF05-17_SV1088, ARKF04-19_SV1088, ARKF04-OZA_SV1096, ARKF04-OZA2_AU0302) and tested it on the two most recent recorders (ARKF04-21_SV1097 and ARKF05-20_SV1391). The results showed similar overall performance to the original CNNs, though with a higher false-positive rate for ARKF05-20_SV1391 (0.02 after partial false-positive control), likely due to an unknown mooring-related sound. Sensitivity was also slightly reduced for ARKF04-21_SV1097 (0.75).

Our CNNs were primarily trained on data from eastern Fram Strait, which lacked complex song and included few biological and cryogenic sounds that could be confused with bowhead whale calls. This made CNN application highly effective for analyzing the large dataset from Fram Strait, reducing the time needed for both training and false positive control to a few days. In contrast, the limited amount of training data for the recorder northwest of Svalbard (ARKY5-01_SV1101) made false-positive control for this position more demanding. The control showed that the CNN could still detect complex song, but struggled to distinguish bowhead whale sounds from those of bearded seals (*Erignathus barbatus*) and ice noises. Despite this, manual song analysis for ARKY5-01_SV1101 (see below) confirmed that nearly all detections from November to February (time period where no false-positive control was applied) were true, likely due to the near-continuous acoustic presence of bowhead whales during this period. The differing effort required for false-positive control at the two recording locations highlights the decisive role of training data availability in CNN applications.

### Song analysis

To analyze the amount and temporal occurrence of songs northwest of Svalbard, we randomly selected one file per hour from final detections of ARKY5-01_SV1101 and inspected them in Raven Pro 1.6.4, using spectrogram settings optimized per frequency range of interest. We distinguished songs based on the presence of markedly distinct units and unit combinations, with structurally similar units considered variations of one another. We chose this definition because several songs exhibited gradual development, with progressively changing unit structures that made it difficult to clearly separate new songs from variations of previous ones. We further allowed for varying unit repetition rates within songs, in line with previous studies^27,33^. Songs were labeled numerically in the order of their first occurrence.

### Environmental data

Daily sea-ice concentration with a resolution of 3.125 × 3.125 km^64^ was sourced from the University of Bremen (https://data.seaice.uni-bremen.de/amsr2/asi_daygrid_swath/n3125/). Sea-ice concentration was averaged within a 35 km radius around each recorder position, in accordance with estimations of maximum sound propagation ranges for bowhead whale vocalizations^22,65^. The distance to the ice edge was determined by calculating the shortest distance between the recorder’s position and the nearest sea ice (≥ 15% concentration) when in open water or to the nearest open water when in ice-covered waters (< 15% concentration). Ice or water patches (≤ 50 pixels, equivalent to ca. 488 km^2^) were excluded from distance calculations.

## Supplementary Information

Supplementary Material 1

## Data Availability

Passive acoustic monitoring data are available from the PANGAEA database (10.1594/PANGAEA.967562, 10.1594/PANGAEA.967551, 10.1594/PANGAEA.967512, 10.1594/PANGAEA.967475, 10.1594/PANGAEA.956286, 10.1594/PANGAEA.967557, 10.1594/PANGAEA.967489, 10.1594/PANGAEA.986815), with additional datasets currently under review.
