# Supplementary material for: Differences in acoustic presence and vocal behavior of Spitsbergen’s bowhead whales under ice-covered and open-water conditions
Source: Sci Rep. 2025 Nov 18;15:37752. doi: 10.1038/s41598-025-25360-2 (PMC12627553; doi:10.1038/s41598-025-25360-2)
Supplement: Supplementary file 1 — Supplementary Material 1 [file 41598_2025_25360_MOESM1_ESM.pdf]

Supplementary material

The following supplement accompanies the article:

Title: *Differences in acoustic presence and vocal behavior of Spitsbergen's bowhead whales under ice-covered and open-water conditions*

Authors: Marlene Meister, Paul Keil, Karolin Thomisch

Table S1: Recorders in the sample dataset and their use in convolutional neural network (CNN) training. X = included in training; CNN_Ea_Ce = model trained on data from eastern/central Fram Strait and applied to data from eastern/central Fram Strait; CNN_No = model trained on data from northwest of Svalbard and from eastern/central Fram Strait, and applied to data from northwest of Svalbard.

**Deployment IDs of recorders in sample dataset**	**CNN_No**	**CNN_Ea_Ce**
ARKF04-15_SV1026	X	X
ARKF16-09_SV1021	X	X
ARKF05-17_SV1088	X	X
ARKF04-OZA_SV1096	X	X
ARKF04-19_SV1088	X	X
ARKF04-OZA2_AU0302	X	X
ARKF04-21_SV1097	X	X
ARKF05-20_SV1391	X	X
ARKY5-01_SV1101	X	

Table S2: Model training parameters of two custom-built sequential CNNs (CNN_No, CNN_Ea_Ce) used for the detection of bowhead whale vocalizations.

**Parameter**	**Value**
Optimizer	Adam
Learning rate	$2e^{-4}$
Weight decay	$1e^{-6}$
Batch size	64
Class weights (CNN_Ea_Cea)	~0.5 to ~11
Class weights (CNN_No)	~0.6 to ~5

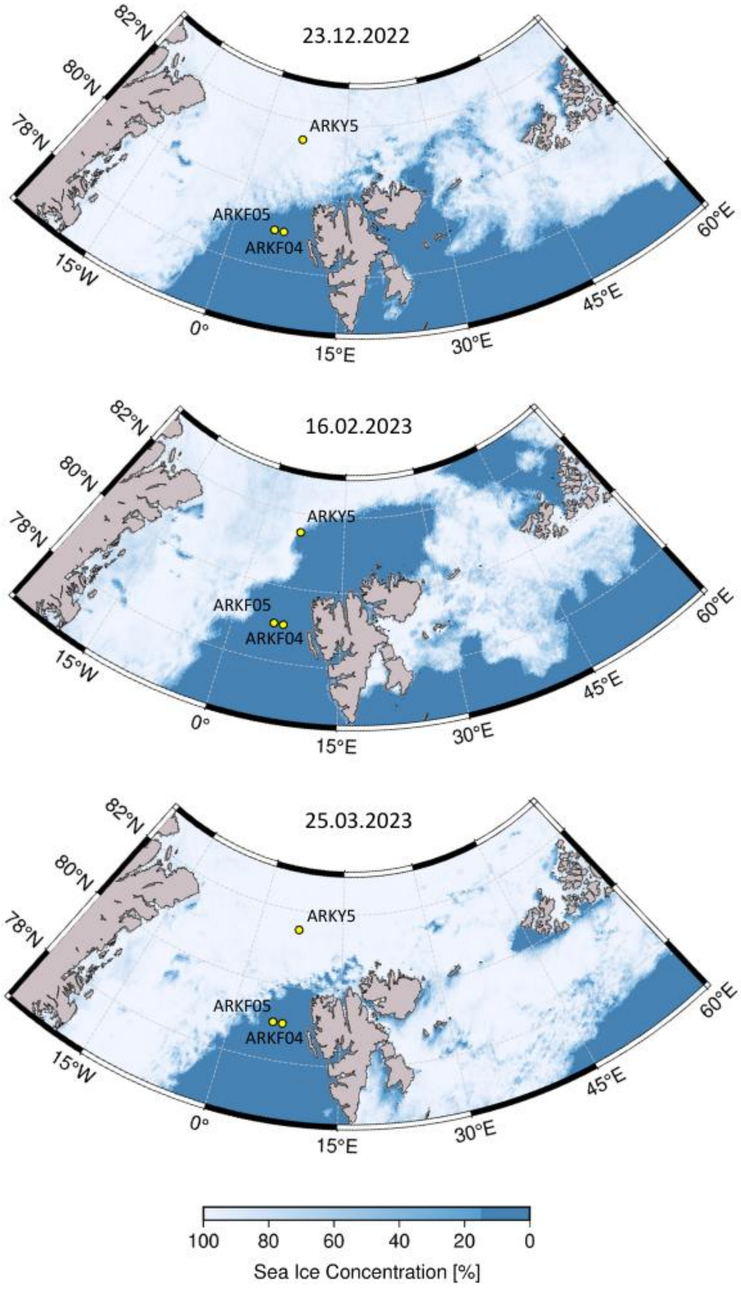

Figure S1: Sea-ice concentration at a selected day in December (upper map) and February (middle map) and March (lower map). Sea-ice concentration with a resolution of 3.125 x 3.125 km (Spreen et al., 2008) was sourced from the University of Bremen (https://data.seaice.uni-bremen.de/amsr2/asi_daygrid_swath/n3125/).

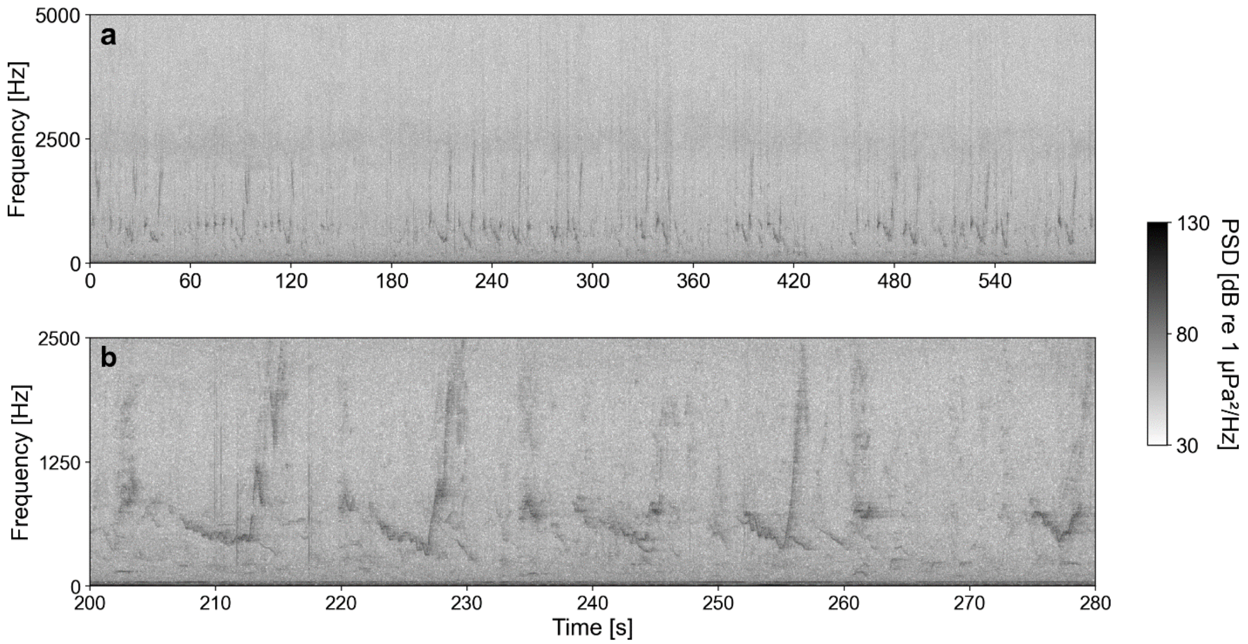

Figure S2: Example of song 1 recorded by ARKY5-01_SV1101 (moored at 81.50°N, 7.15°E) on 18.10.2022. (a) Full 10-minute file; (b) 80-second zoom-in. PSD = Power Spectral Density.

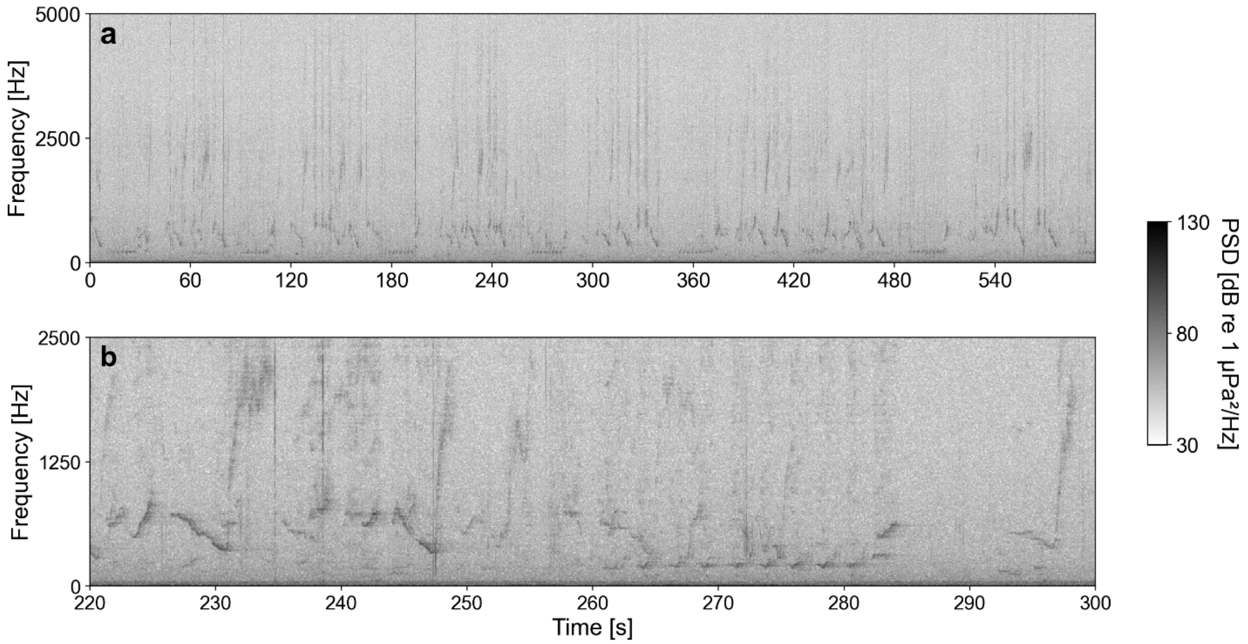

Figure S3: Example of song 1 recorded by ARKY5-01_SV1101 (moored at 81.50°N, 7.15°E) on 19.10.2022. (a) Full 10-minute file; (b) 80-second zoom-in. PSD = Power Spectral Density.

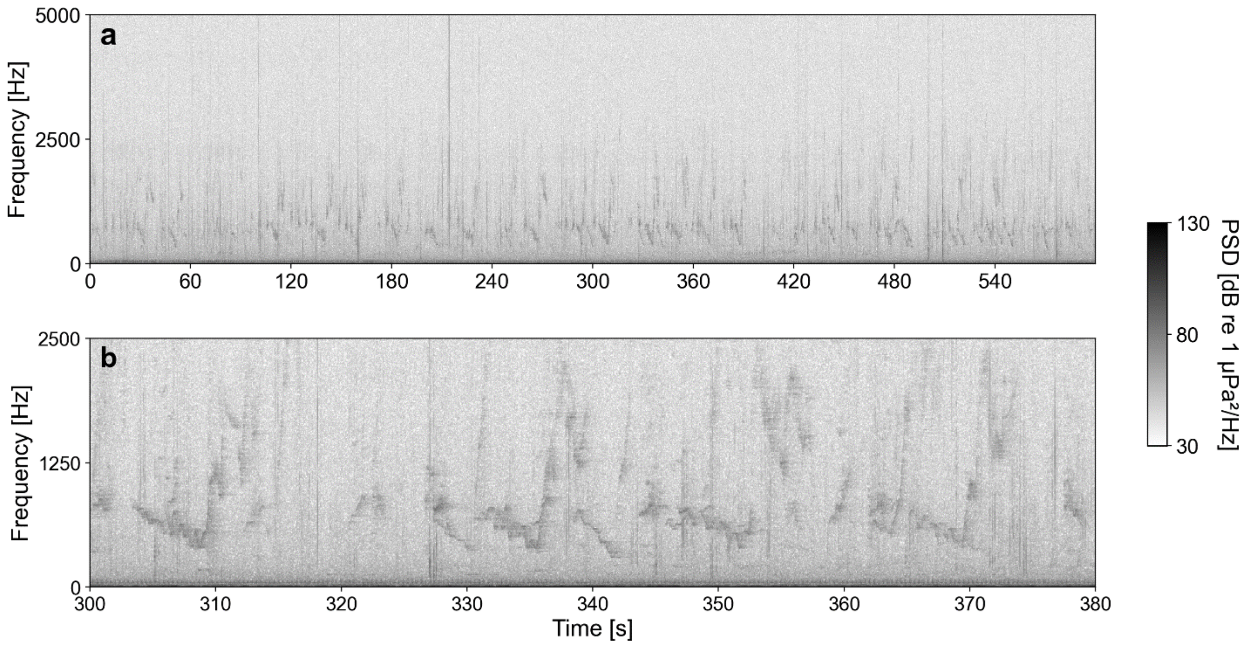

Figure S4: Example of song 1 recorded by ARKY5-01_SV1101 (moored at 81.50°N, 7.15°E) on 26.10.2022. (a) Full 10-minute file; (b) 80-second zoom-in. PSD = Power Spectral Density.

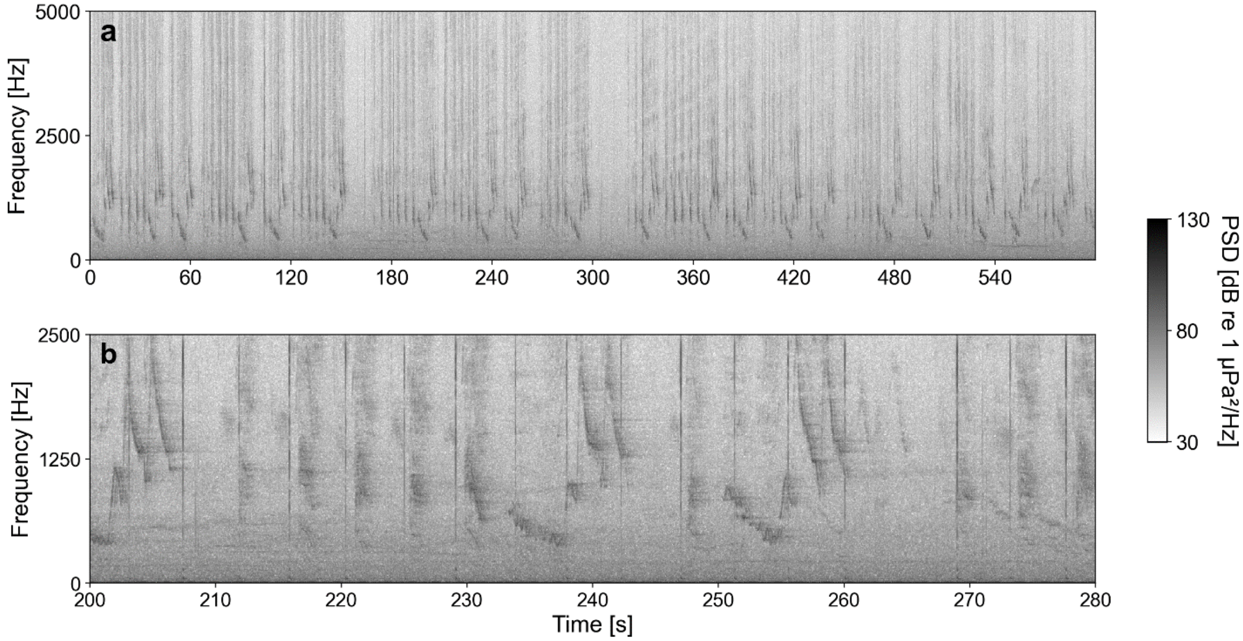

Figure S5: Example of song 1 recorded by ARKY5-01_SV1101 (moored at 81.50°N, 7.15°E) on 20.11.2022. (a) Full 10-minute file; (b) 80-second zoom-in. PSD = Power Spectral Density.

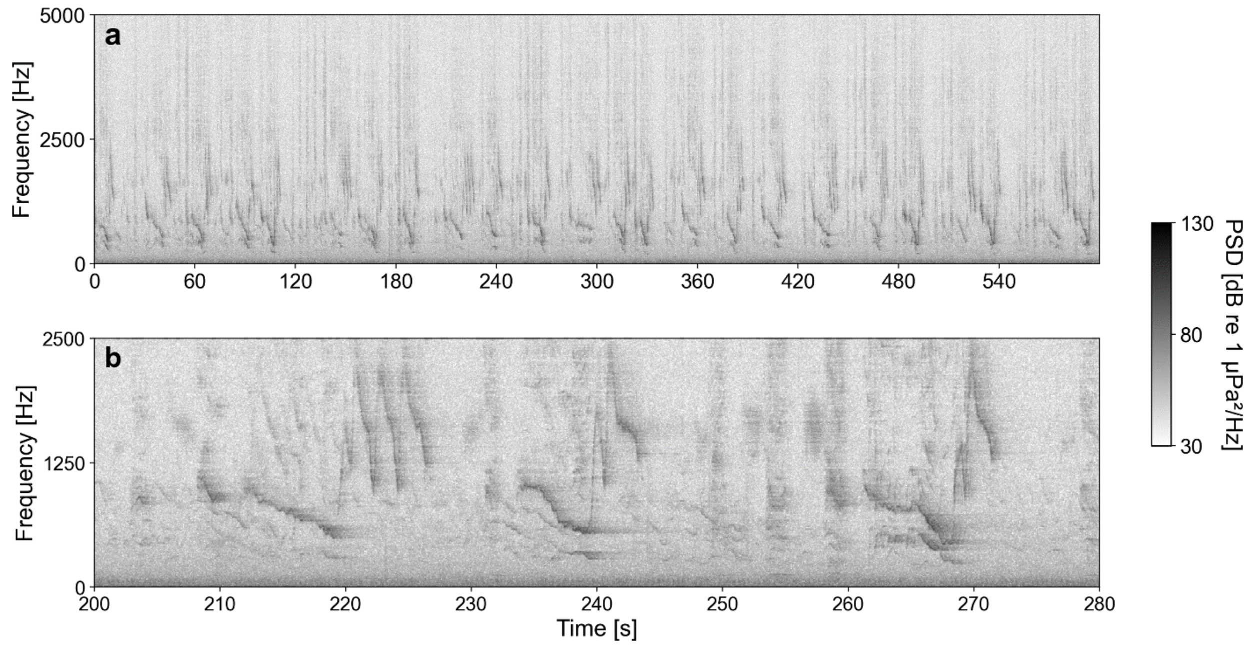

Figure S6: Example of song 1 recorded by ARKY5-01_SV1101 (moored at 81.50°N, 7.15°E) on 21.11.2022. (a) Full 10-minute file; (b) 80-second zoom-in. PSD = Power Spectral Density.

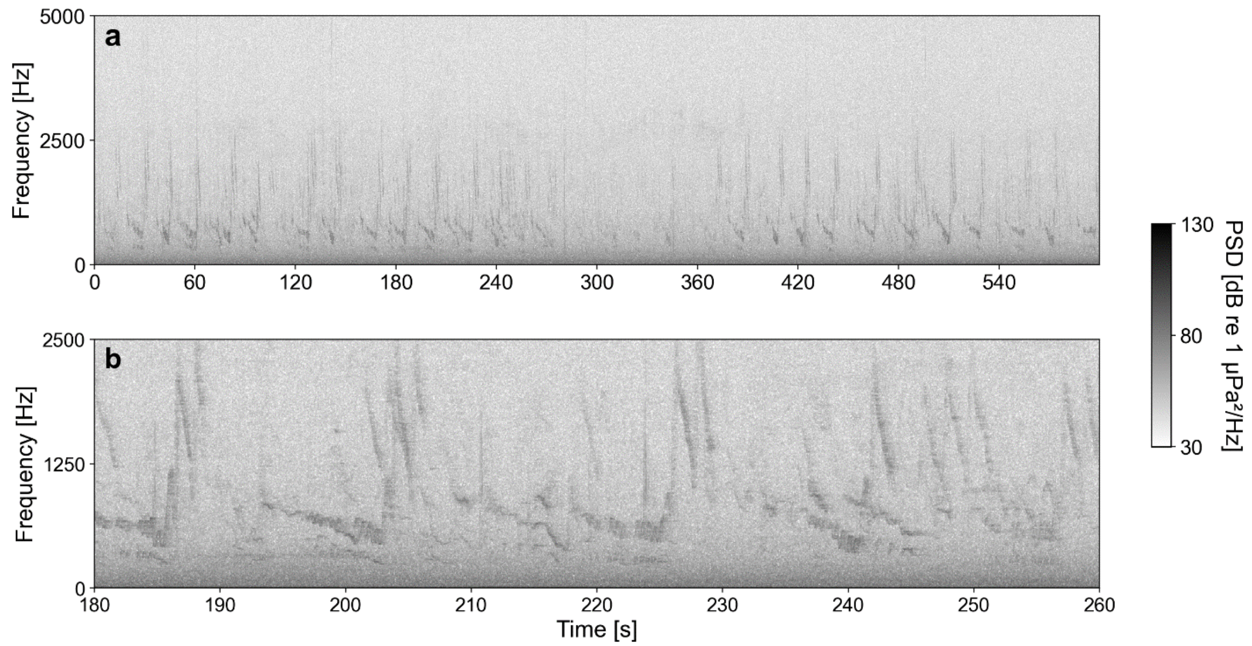

Figure S7: Example of song 1 recorded by ARKY5-01_SV1101 (moored at 81.50°N, 7.15°E) on 27.11.2022. (a) Full 10-minute file; (b) 80-second zoom-in. PSD = Power Spectral Density.

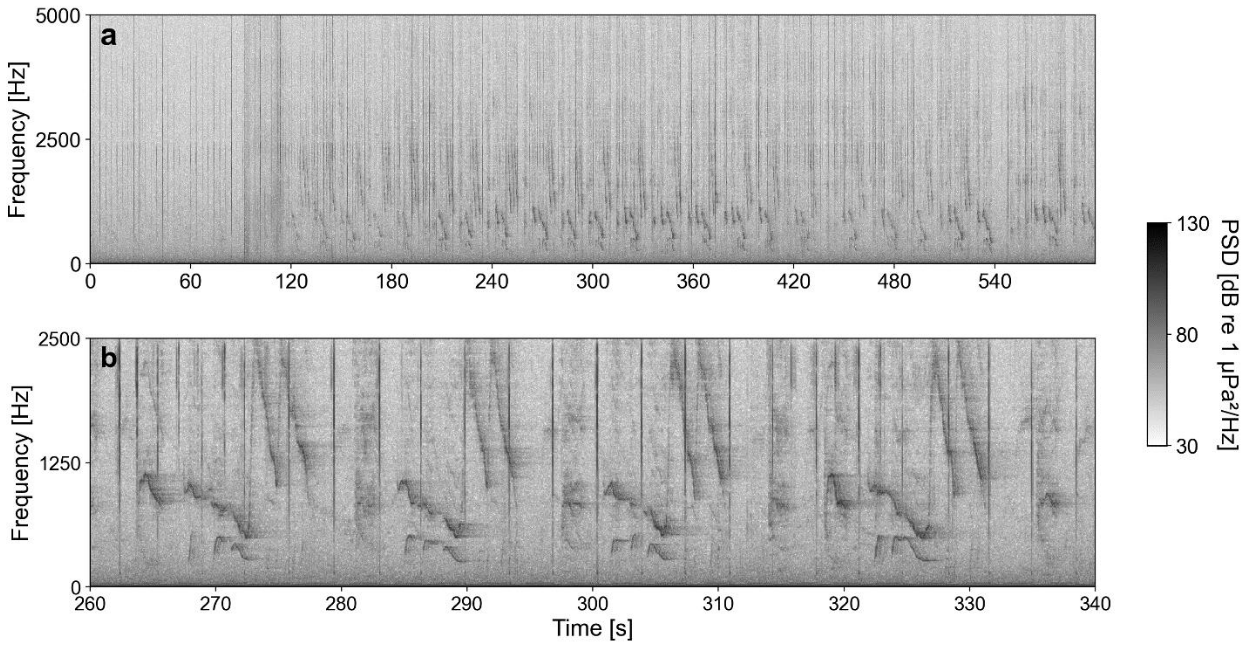

Figure S8: Example of song 1 recorded by ARKY5-01_SV1101 (moored at 81.50°N, 7.15°E) on 29.11.2022. (a) Full 10-minute file; (b) 80-second zoom-in. PSD = Power Spectral Density.

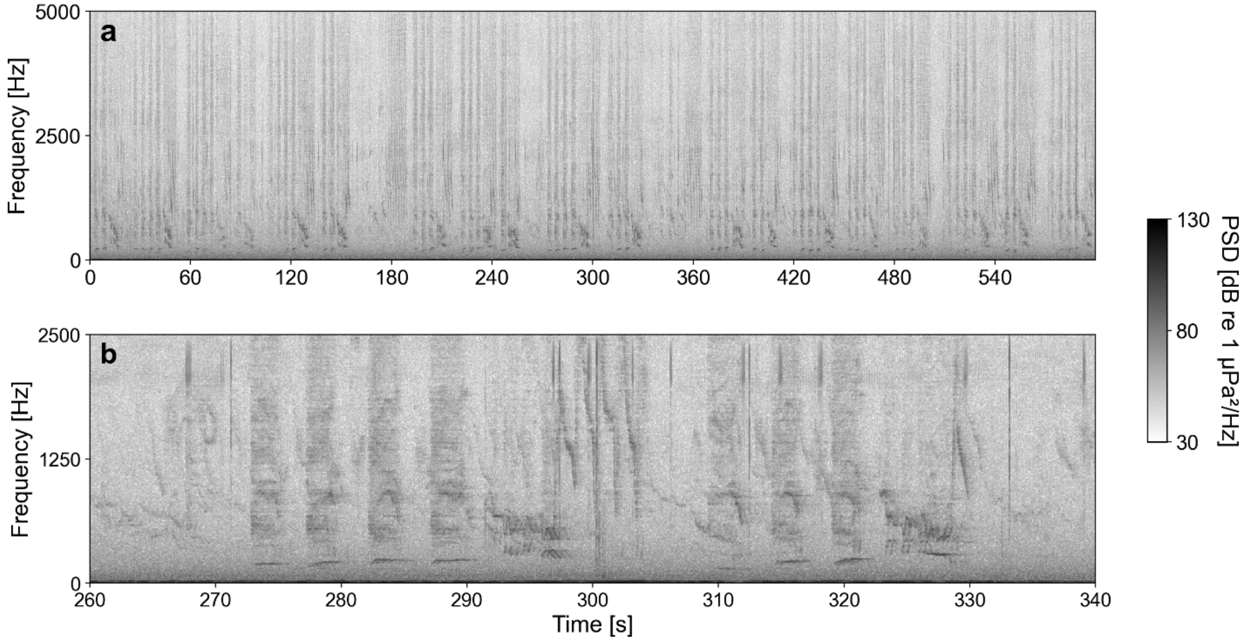

Figure S9: Example of song 1 recorded by ARKY5-01_SV1101 (moored at 81.50°N, 7.15°E) on 29.11.2022. (a) Full 10-minute file; (b) 80-second zoom-in. PSD = Power Spectral Density.

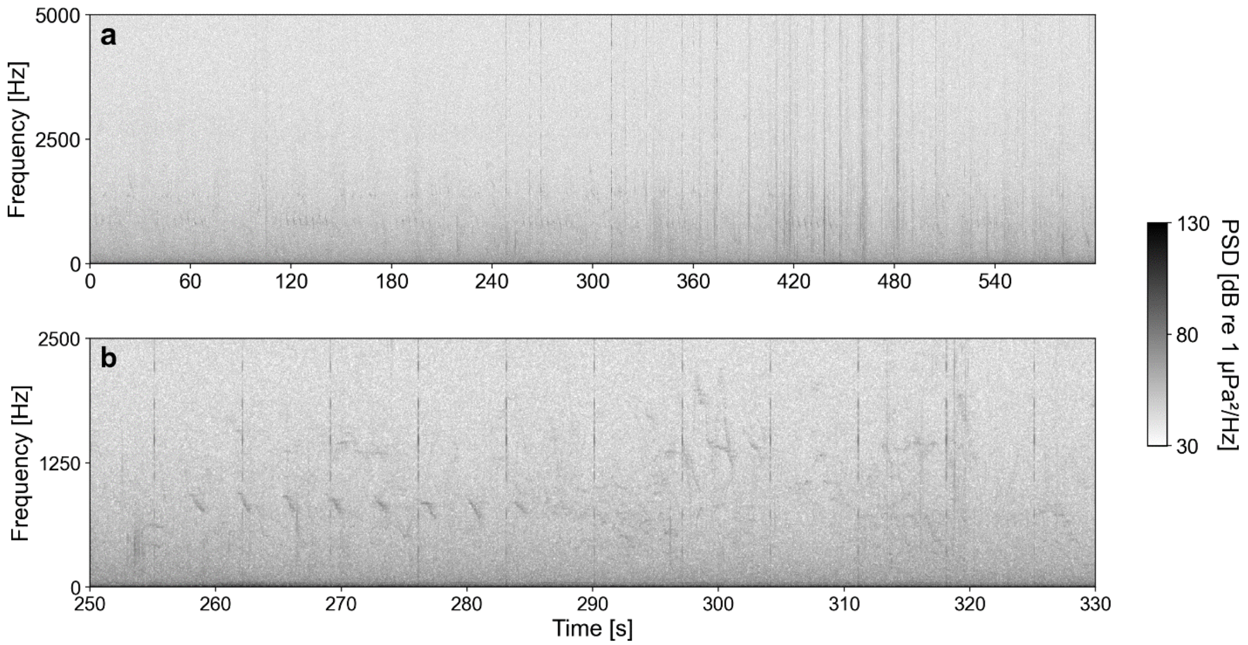

Figure S10: Example of song 2 recorded by ARKY5-01_SV1101 (moored at 81.50°N, 7.15°E) on 25.11.2022. (a) Full 10-minute file; (b) 80-second zoom-in. PSD = Power Spectral Density.

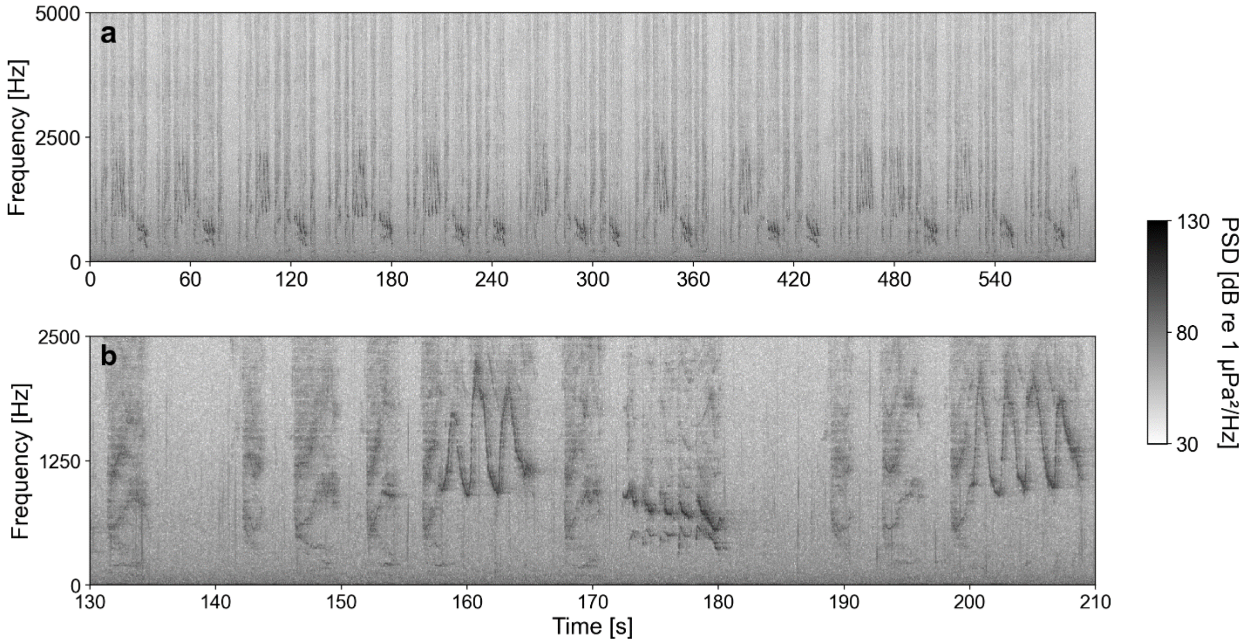

Figure S11: Example of song 3 recorded by ARKY5-01_SV1101 (moored at 81.50°N, 7.15°E) on 02.12.2022. (a) Full 10-minute file; (b) 80-second zoom-in. PSD = Power Spectral Density.

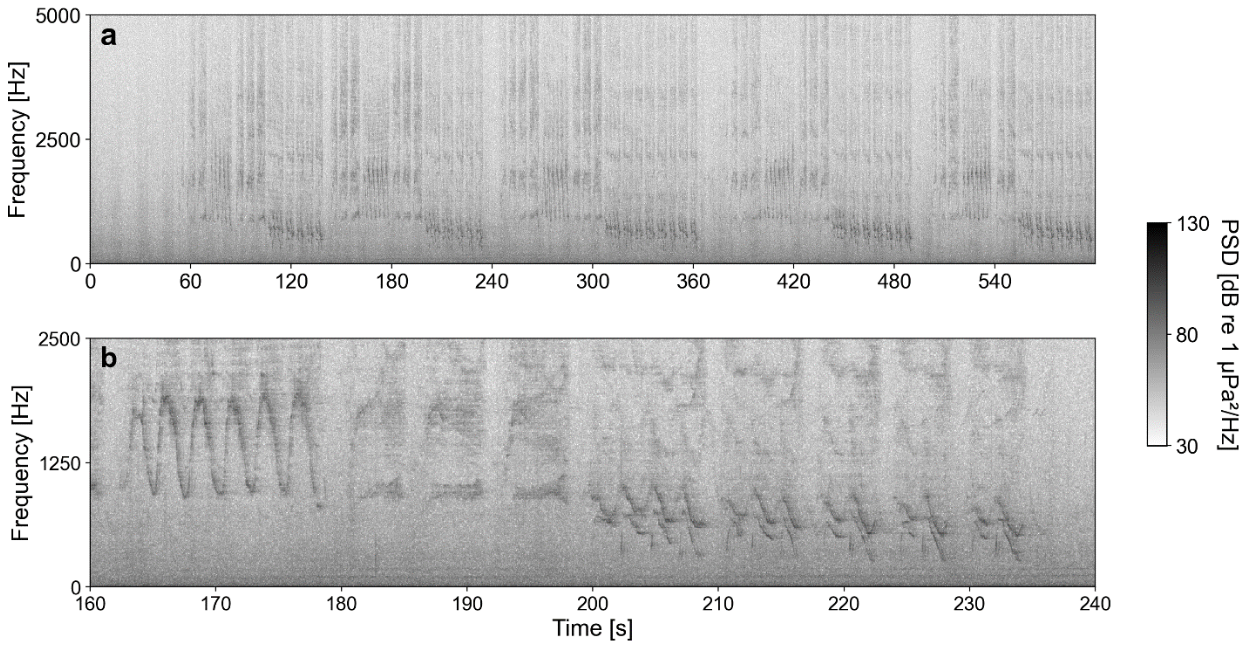

Figure S12: Example of song 3 recorded by ARKY5-01_SV1101 (moored at 81.50°N, 7.15°E) on 31.12.2022. (a) Full 10-minute file; (b) 80-second zoom-in. PSD = Power Spectral Density.

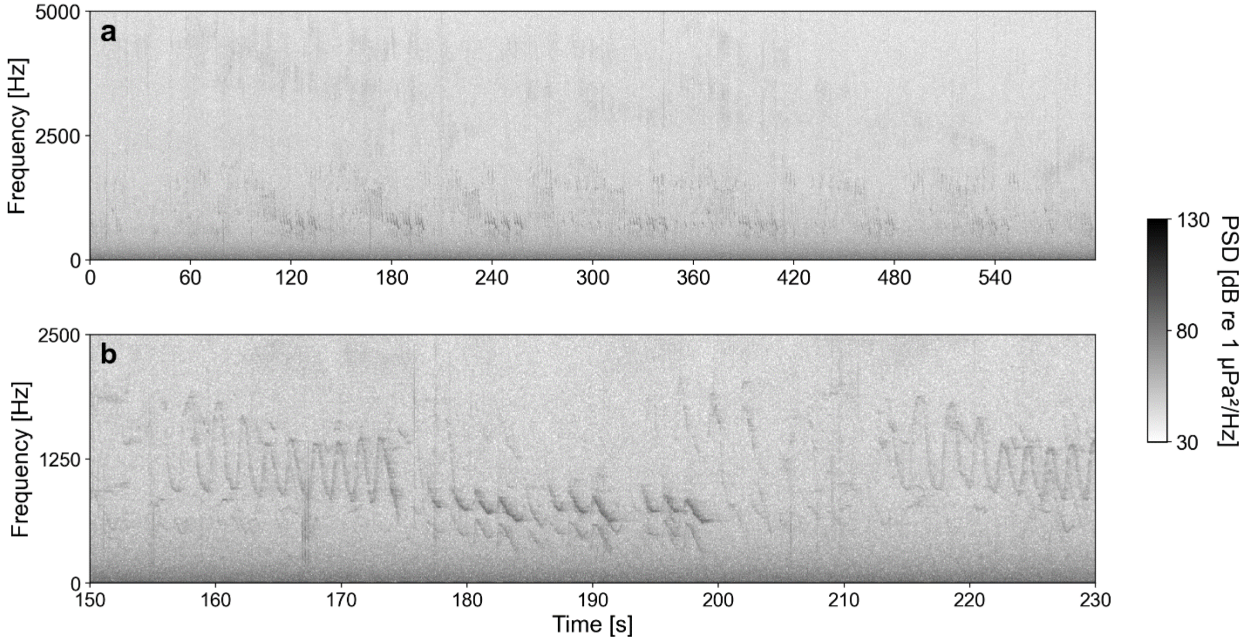

Figure S13: Example of song 3 recorded by ARKY5-01_SV1101 (moored at 81.50°N, 7.15°E) on 03.01.2023. (a) Full 10-minute file; (b) 80-second zoom-in. PSD = Power Spectral Density.

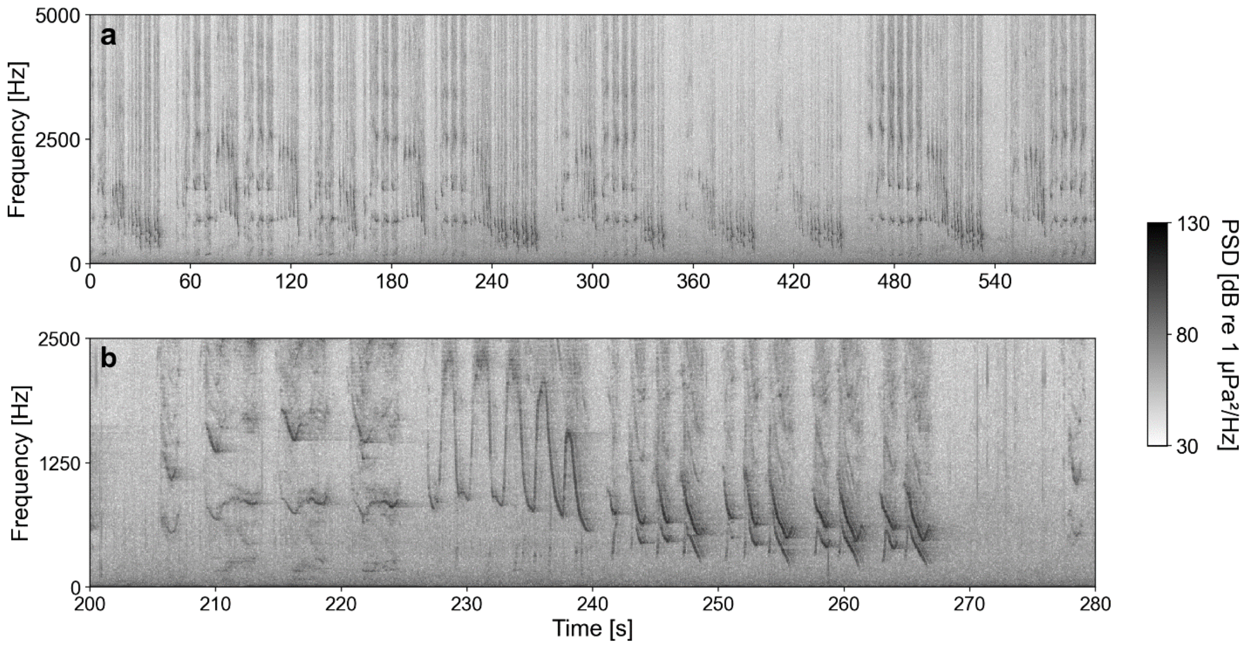

Figure S14: Example of song 3 recorded by ARKY5-01_SV1101 (moored at 81.50°N, 7.15°E) on 04.01.2023. (a) Full 10-minute file; (b) 80-second zoom-in. PSD = Power Spectral Density.

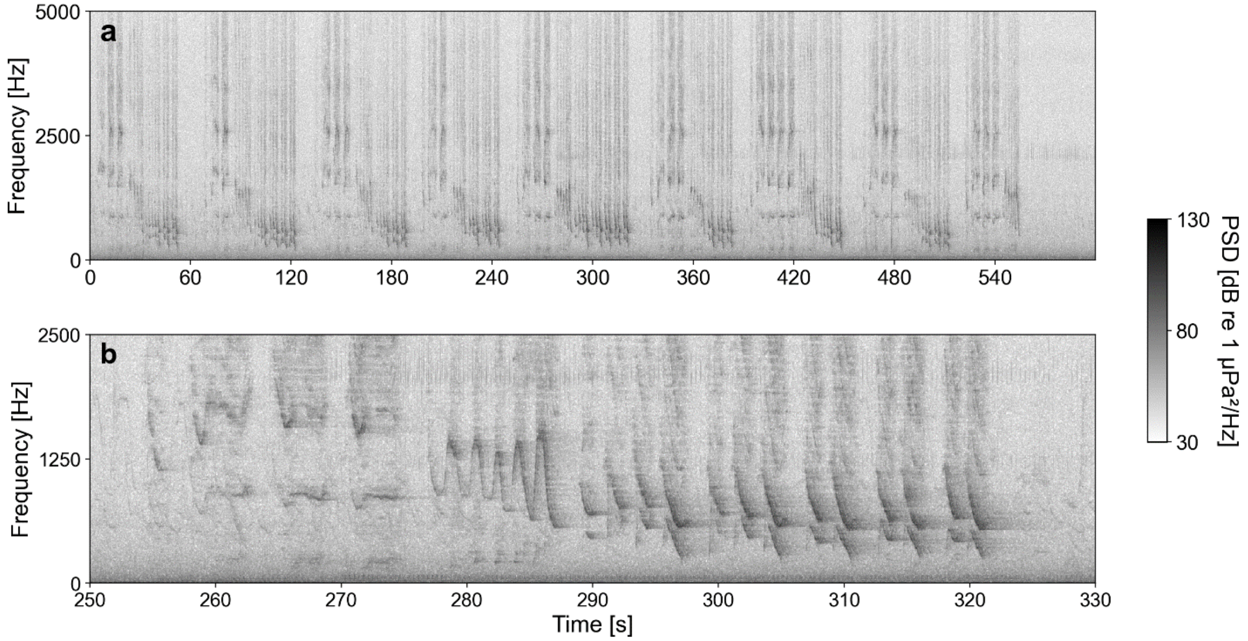

Figure S15: Example of song 3 recorded by ARKY5-01_SV1101 (moored at 81.50°N, 7.15°E) on 05.01.2023. (a) Full 10-minute file; (b) 80-second zoom-in. PSD = Power Spectral Density.

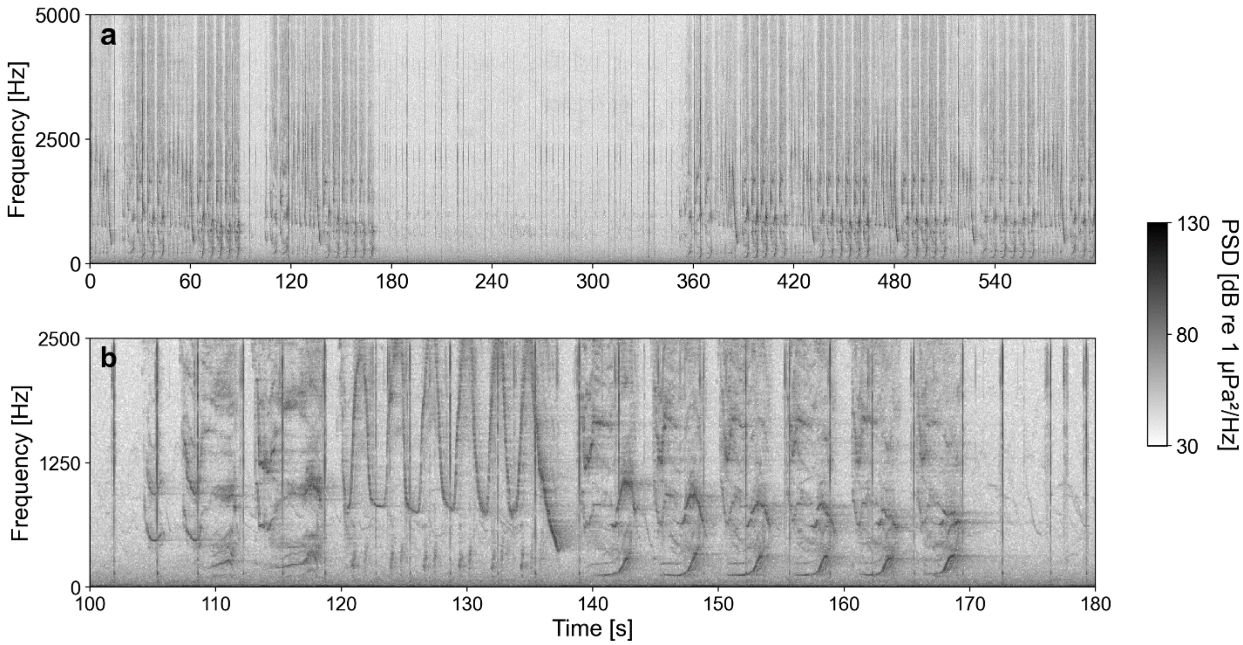

Figure S16: Example of song 4 recorded by ARKY5-01_SV1101 (moored at 81.50°N, 7.15°E) on 04.01.2023. (a) Full 10-minute file; (b) 80-second zoom-in. PSD = Power Spectral Density.

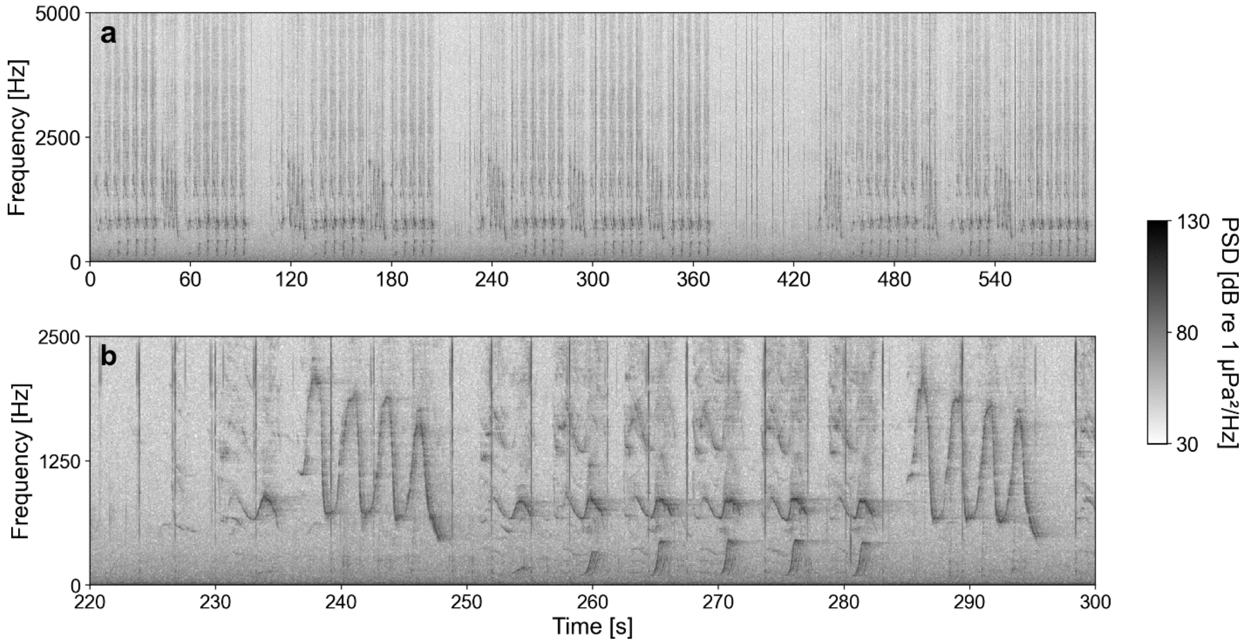

Figure S17: Example of song 4 recorded by ARKY5-01_SV1101 (moored at 81.50°N, 7.15°E) on 25.01.2023. (a) Full 10-minute file; (b) 80-second zoom-in. PSD = Power Spectral Density.

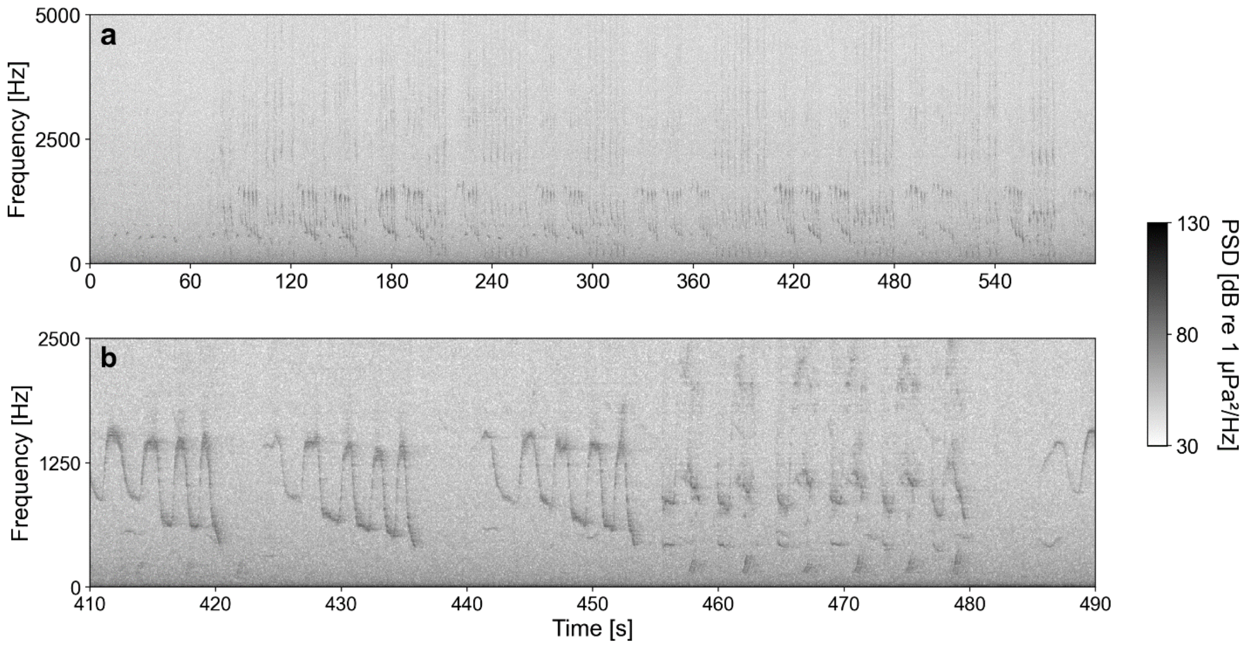

Figure S18: Example of song 4 recorded by ARKY5-01_SV1101 (moored at 81.50°N, 7.15°E) on 21.02.2023. (a) Full 10-minute file; (b) 80-second zoom-in. PSD = Power Spectral Density.

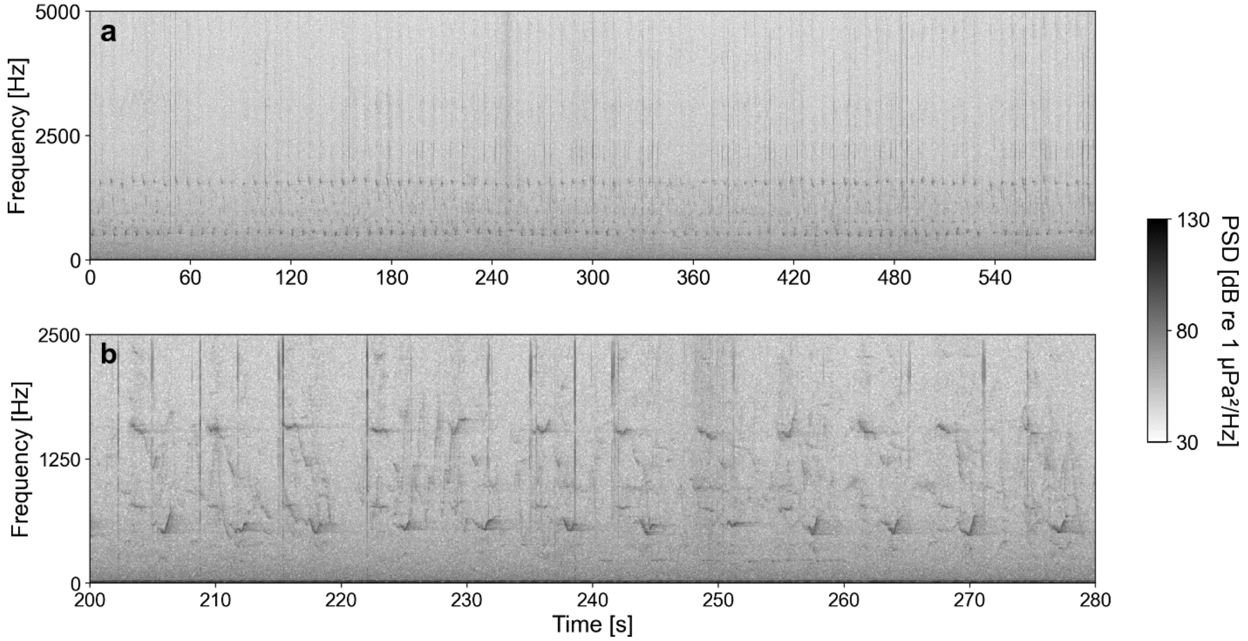

Figure S19: Example of song 5 recorded by ARKY5-01_SV1101 (moored at 81.50°N, 7.15°E) on 12.01.2023. (a) Full 10-minute file; (b) 80-second zoom-in. PSD = Power Spectral Density.

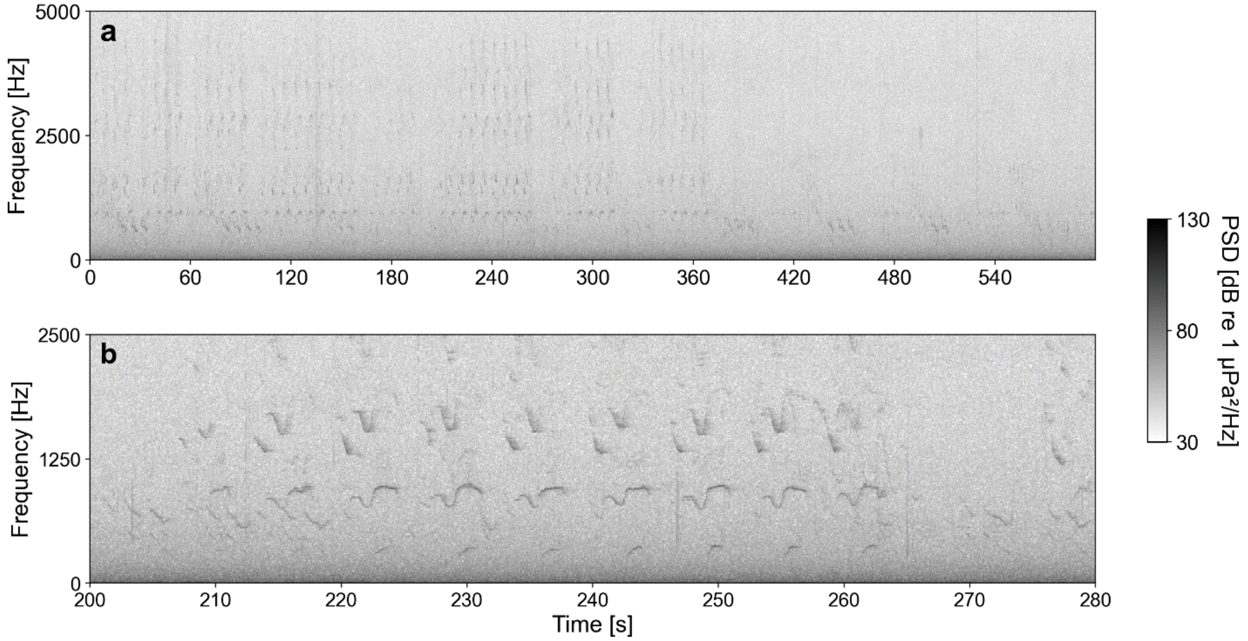

Figure S20: Example of song 5 recorded by ARKY5-01_SV1101 (moored at 81.50°N, 7.15°E) on 23.01.2023. (a) Full 10-minute file; (b) 80-second zoom-in. PSD = Power Spectral Density.

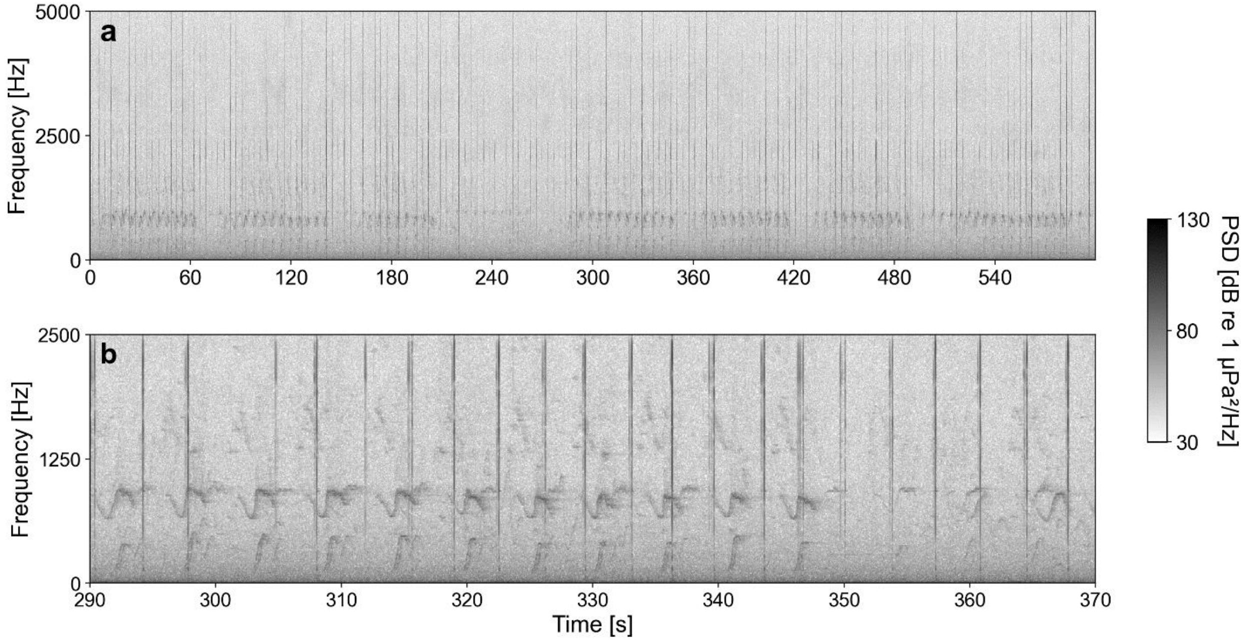

Figure S21: Example of song 5 recorded by ARKY5-01_SV1101 (moored at 81.50°N, 7.15°E) on 27.01.2023. (a) Full 10-minute file; (b) 80-second zoom-in. PSD = Power Spectral Density.

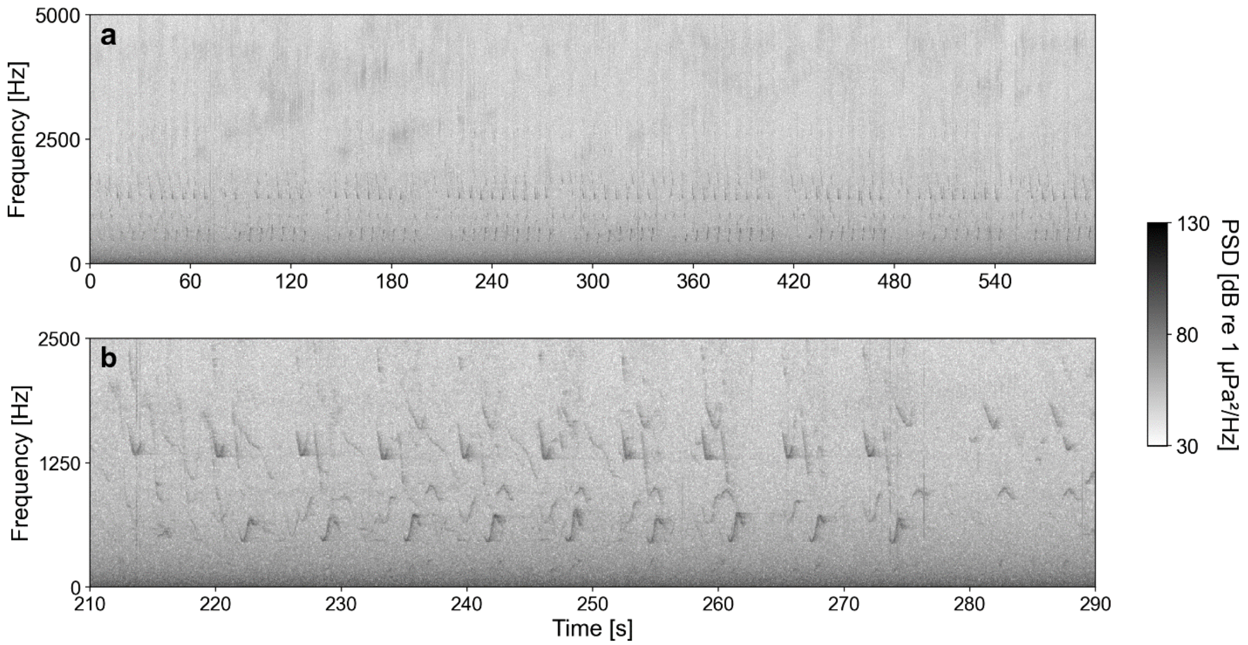

Figure S22: Example of song 5 recorded by ARKY5-01_SV1101 (moored at 81.50°N, 7.15°E) on 07.02.2023. (a) Full 10-minute file; (b) 80-second zoom-in. PSD = Power Spectral Density.

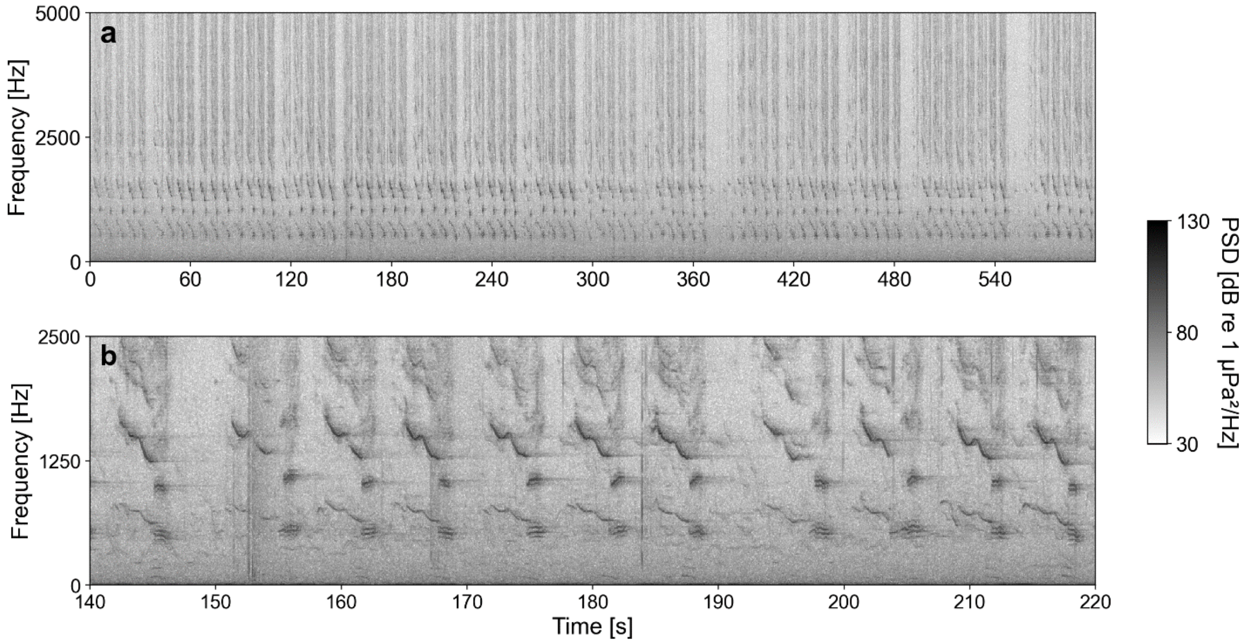

Figure S23: Example of song 6 recorded by ARKY5-01_SV1101 (moored at 81.50°N, 7.15°E) on 09.01.2023. (a) Full 10-minute file; (b) 80-second zoom-in. PSD = Power Spectral Density.

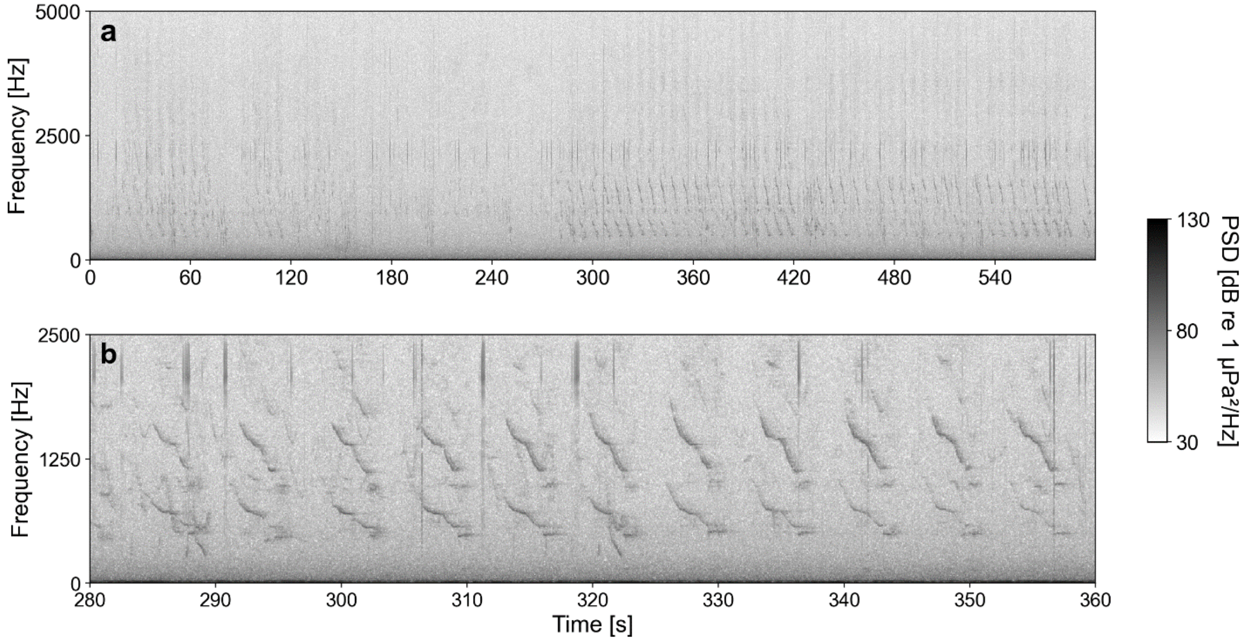

Figure S24: Example of song 6 recorded by ARKY5-01_SV1101 (moored at 81.50°N, 7.15°E) on 23.01.2023. (a) Full 10-minute file; (b) 80-second zoom-in. PSD = Power Spectral Density.

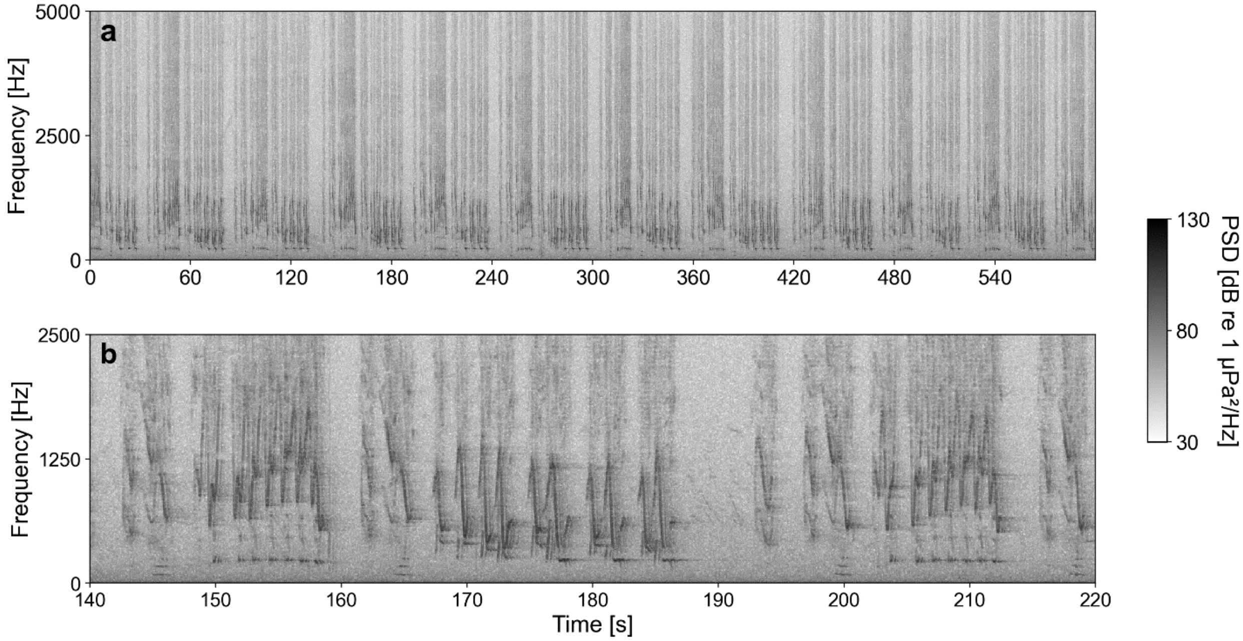

Figure S25: Example of song 7 recorded by ARKY5-01_SV1101 (moored at 81.50°N, 7.15°E) on 12.01.2023. (a) Full 10-minute file; (b) 80-second zoom-in. PSD = Power Spectral Density.

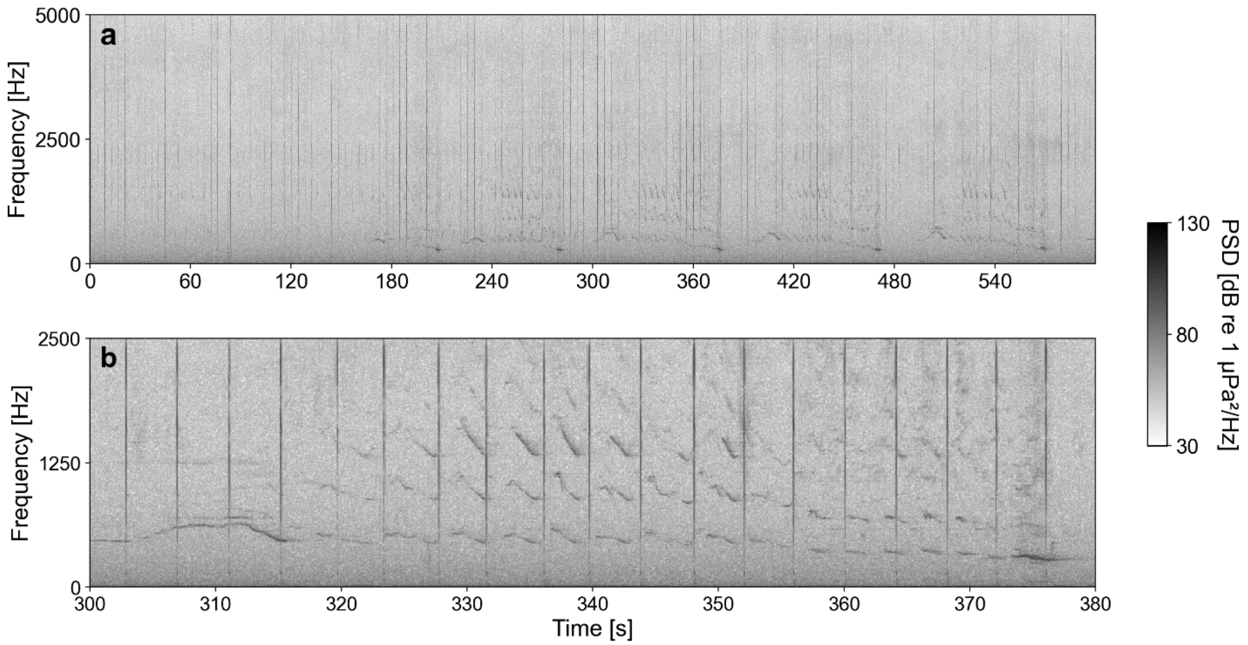

Figure S26: Example of song 8 recorded by ARKY5-01_SV1101 (moored at 81.50°N, 7.15°E) on 11.02.2023. (a) Full 10-minute file; (b) 80-second zoom-in. PSD = Power Spectral Density.

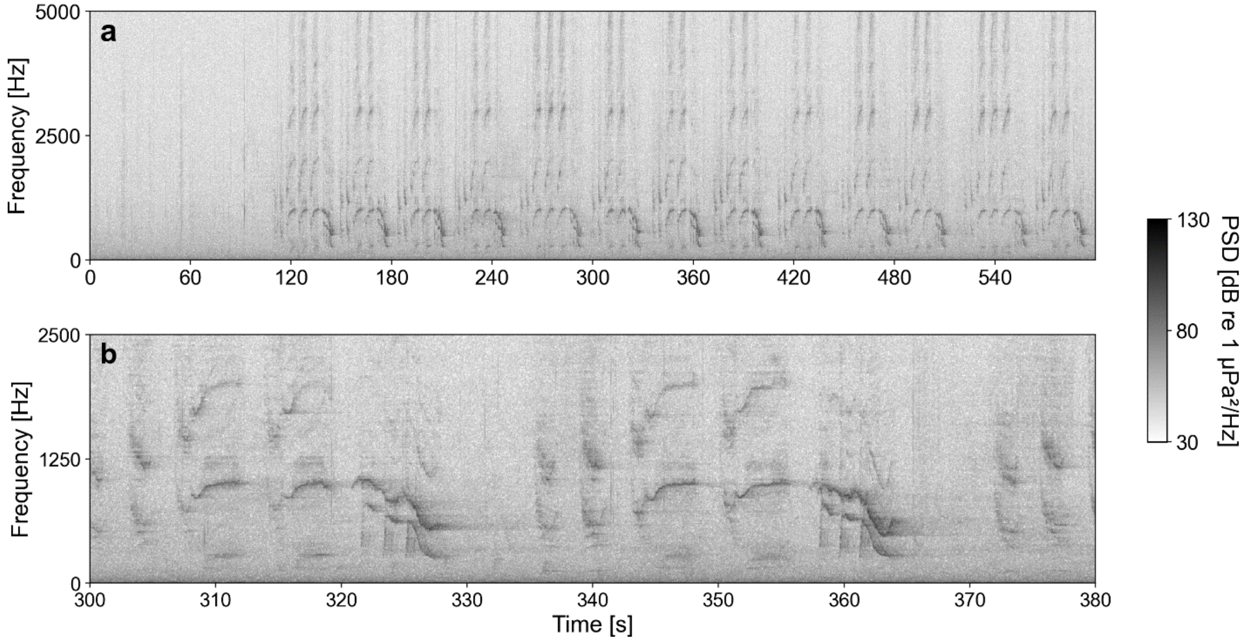

Figure S27: Example of song 9 recorded by ARKY5-01_SV1101 (moored at 81.50°N, 7.15°E) on 20.02.2023. (a) Full 10-minute file; (b) 80-second zoom-in. PSD = Power Spectral Density.

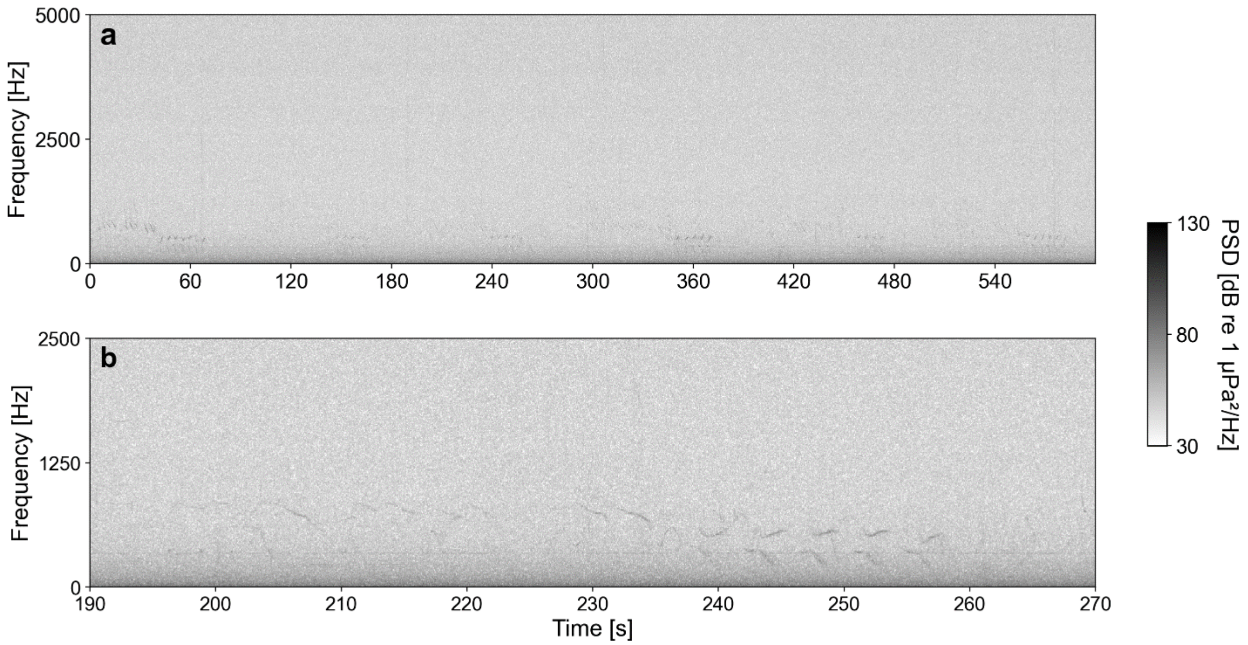

Figure S28: Example of song 10 recorded by ARKY5-01_SV1101 (moored at 81.50°N, 7.15°E) on 27.03.2023. (a) Full 10-minute file; (b) 80-second zoom-in. PSD = Power Spectral Density.

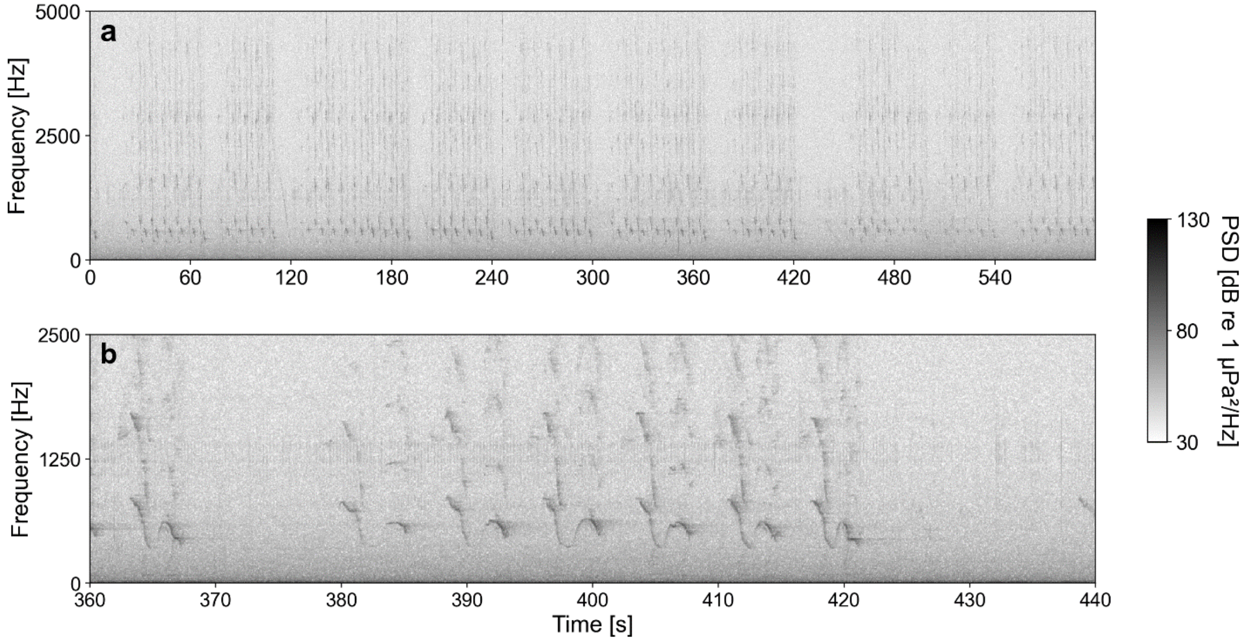

Figure S29: Example of song 11 recorded by ARKY5-01_SV1101 (moored at 81.50°N, 7.15°E) on 29.03.2023. (a) Full 10-minute file; (b) 80-second zoom-in. PSD = Power Spectral Density.

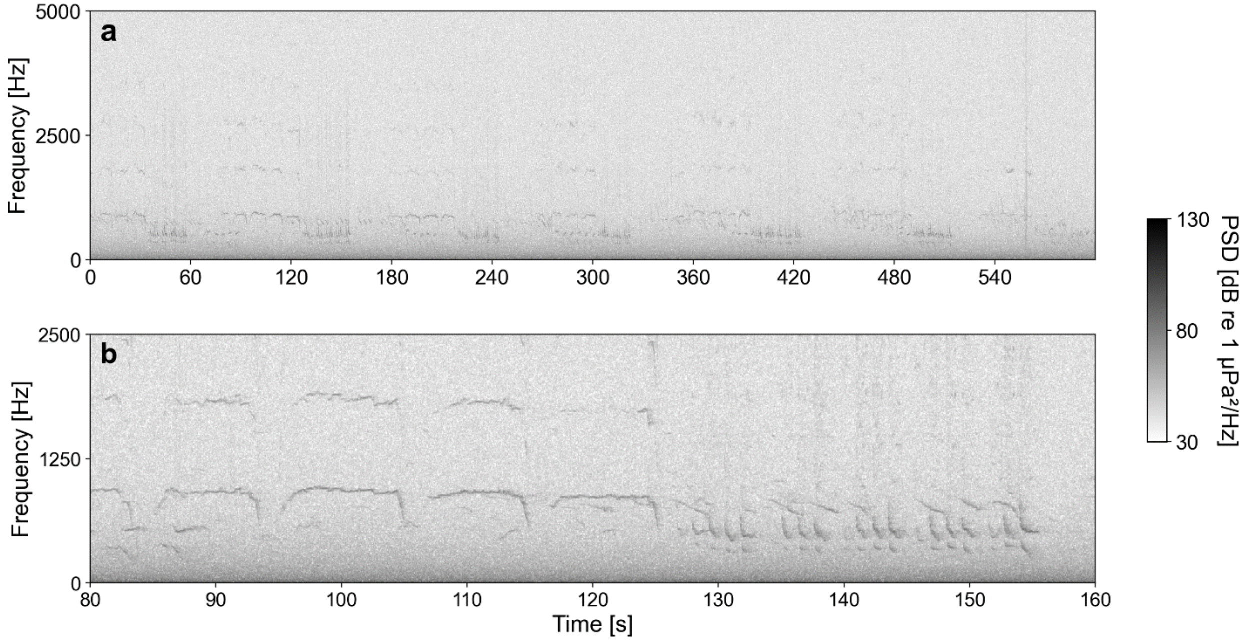

Figure S30: Example of song 12 recorded by ARKY5-01_SV1101 (moored at 81.50°N, 7.15°E) on 24.03.2023. (a) Full 10-minute file; (b) 80-second zoom-in. Song 10 is also partially visible, overlapping with song 12. PSD = Power Spectral Density.
